# Supplementary material for: Genome-resolved biogeography of Phaeocystales, cosmopolitan bloom-forming algae
Source: Nat Commun. 2025 Sep 29;16:8559. doi: 10.1038/s41467-025-63565-1 (PMC12480563; doi:10.1038/s41467-025-63565-1)
Supplement: Supplementary file 1 — Supplementary Information [file 41467_2025_63565_MOESM1_ESM.pdf]

SUPPLEMENTARY INFORMATION FOR:

Genome-resolved biogeography of Phaeocystales, cosmopolitan bloom-forming microalgae

Zoltán Füssy, Robert H. Lampe, Kevin R. Arrigo, Kerrie Barry, Margaret M. Brisbin, Corina P. D. Brussaard, Johan Decelle, Colomban de Vargas, Giacomo R. DiTullio, Liam D. H. Elbourne, Marc E. Frischer, David M. Goodstein, Igor V. Grigoriev, Richard D. Hayes, Adam L. Healey, Chase C. James, Jerry Jenkins, Caroline Juery, Manish Kumar, Adam B. Kustka, Florian Maumus, Anna MG Novák Vanclová, Miroslav Oborník, Ian T. Paulsen, Ian Probert, Mak A. Saito, Jeremy Schmutz, Tomáš Skalický, Diego Tec-Campos, Hannah Tomelka, Pavlína Věchtová, Pratap Venepally, Brendan Wilson-Mortier, Karsten Zengler, Hong Zheng, Andrew E. Allen

Contents:

Abbreviations

Supplementary Notes

1. Genome assembly; 2. Repetitive elements; 3. Genome-assisted biogeography; 4. Functional annotation; 5. Physiological responses of Phaeocystales in polar and temperate oceans; 6. Gene family expansion and horizontal gene transfer; 7. Transcriptomic analyses of *P. globosa*;

Supplementary Data 1-10 legends

Supplementary Figures 1-8

Abbreviations: CalCOFI (NCOG) California Cooperative Oceanic Fisheries Investigations (NOAA-CalCOFI Ocean Genomics) Project, CCE – California Current Ecosystem, CICLOPS - Cobalamin and Iron Co-Limitation of Phytoplankton Species Project, DCM – deep chlorophyll maximum, GAM – Generalized Additive Models, GEM – genome-scale metabolic model, HGT – horizontal gene transfer, hpi – hours post-infection, KOG – euKaryotic Orthologous Groups, KOs – KEGG orthologs, MAG – metagenome-assembled genome, metaG/metaT – metagenomic/metatranscriptomic, mCP/MCP – minor/major capsid protein, MMETSP – the Marine Microbial Eukaryotic Transcriptome Sequencing Project, (Pae/Pge)NCLDV – (Phaeocystis antarctica/globosa endogenous) nucleo-cytoplasmic large DNA virus, OGs – orthologous groups, ORF – open reading frame, PgV – Phaeocystis globosa virus, PgVV – Phaeocystis globosa virus virophage, (Pe)PLV – (Phaeocystis endogenous) Polinton-like virus, PSC – *Phaeocystis* sister clade, RPM – reads per million, SOM – Self-Organizing Maps, TEs – transposable elements, TPM – transcripts per million.

SUPPLEMENTARY NOTES

Supplementary Note 1.

Genome assembly

The assemblies' sizes ranged 86.7-199.1 Mbp, while the expected true genome sizes range from 71.8 Mbp (*P. cordata* CCMP3104) to over 196 Mbp (*P. rex* CCMP2000), which could be a result of insufficient genome read coverage in the latter case. The size of the recently published chromosome-level genome assembly of *P. globosa* strain CNS00066 (a clade/genotype different from the ones presented here) is 129.7 Mbp[1]. The draft genomes of most genotypes are highly fragmented, as apparent from their N50 (Supplementary Data 1). This is despite our effort to remove bacterial contamination and correct/normalize read input (see Material and Methods) and likely stems from the limitations of short read-only data assembly. Indeed, according to GenomeScope 2.0 estimates, substantial portions of the data represent simple repeats (37-69%; not shown). Due to these technical problems and limitations, repetitive regions remain difficult to assemble and quantify in *Phaeocystis* spp., along with recently duplicated genes they might flank. The varying level of assembly quality introduces biases in coding and non-coding repeats, which is why we did not attempt to quantify gene family expansion in the fragmented assemblies. To assess the completeness of culture-based and environmental assemblies, we used the BUSCO and CEGMA platforms and found values comparable to the *Emiliania huxleyi* reference genome (Supplementary Data 1). Taxonomy-informed homology searches were also performed, whereby gene models derived from the best assemblies

Phaant1 and Phaglo1 were queried against each draft genome/MAG and against a haptophyte outgroup database. Most Phaant1 and Phaglo1 models found stronger matches in the respective draft genomes than in the outgroup database, suggesting these models are well represented in our draft genomes and are consistent with the completeness determined by BUSCO and CEGMA (Supplementary Data 1). The 37,567, 33,431, and 29,900 gene annotations for Phaant1, Phacord1, and Phaglo1 are excluding overlapping genes and endogenous virus loci. For comparison, the chromosome-level assembly of *P. globosa* strain CNS00066 has 32,618 gene models predicted[1].

Plastid genomes from cultured strains were assembled to full length (105.5-110.4 kbp; Supplementary Data 1) and have a canonical circular-mapping architecture with two single-copy regions and two inverted repeats (IRs; IR-A and IR-B). They are highly collinear and uniformly carry in the single-copy regions 109 protein-coding gene plus a variable number of unknown open reading frames, resembling other known haptophyte genomes (95.3-105.3 kbp; 111-119 proteins[2]). The IRs contain three ribosomal genes (Supplementary Fig. 1), but IR-A and IR-B are non-identical in all assemblies except *P. jahnii*, as IR-B lost two tRNA genes. Although this has been seen in haptophytes[2,3], in *Phaeocystis* this asymmetry has persisted over an extended evolutionary time of >70 My (main text Fig. 1). Unusually, *Phaeocystis* plastid genomes use an alternative genetic code with UGA encoding Trp (Supplementary Fig. 1; GTG and ATT are sometimes predicted as start, i.e., codon table 4).

The mitochondrial genomes of *Phaeocystis* spp. are much less collinear, indicating frequent rearrangements (Supplementary Fig. 1). These genomes, likely circular-mapping, are 23.5-34.6 kbp long (Supplementary Data 1), and carry 20 protein-coding genes. The assemblies, with inverted repeats at both ends, proved difficult to circularize using short reads before[4]. Overall, they are comparable in size and gene content to other haptophyte mitogenomes (28.6-34.3 kbp, 20-22 protein-coding genes), and repeat elements, as seen in *Phaeocystis*, are also common in haptophytes[2].

Mash distance analysis, which approximates average nucleotide identity[5], supports a phylogenetic split between *P. globosa* accessions identified by phylogenomic analyses, with two valuable insights. First, divergence rates differ for the three genetic compartments with the plastid genome having the lowest and the mitochondrial genome having the highest substitution rates (Supplementary Fig. 1; compare to [6]); second, whereas this holds true between species, for six *P. globosa* accessions—CCMP627, -628, -629, -1524, -1528 and -2710—the nuclear genetic distance is higher than the mitochondrial, suggesting these six represent a single population. *P. globosa* accessions CCMP2754, -1805 and Phaglo1 are somewhat more divergent in terms of mitochondrial

genetic distance from the former group. Specifically, the mitochondrial genomic rearrangements of CCMP2754 suggest there may be cryptic speciation within *P. globosa* (Supplementary Fig. 1). Neither conventional marker genes (18S rDNA, rbcL) nor our smaller 17-gene matrix showed high phylogenetic support for these splits (not shown), offering a reason why this cryptic speciation might have been overlooked.

The 36 metagenome-assembled genomes (MAGs) with phylogenetic affiliation to *Phaeocystis* according to Delmont et al.[7] were placed to various positions within the phylogeny of Phaeocystales (Supplementary Data 1); 11 MAGs branched within the *antarctica*/*globosa*/*pouchetii* species complex and at least three of these belong to the polar clade; 2 MAGs branched closely sister to *P. cordata*; another 11 MAGs branched distantly to the cultivated *Phaeocystis* strains, of which three, two and six formed supported clades with the *antarctica*/*globosa*/*pouchetii* species complex, *P. cordata* and *P. jahnii*, respectively; 9 MAGs branched even more distantly, forming a clade previously referred to as "sister *Phaeocystis*"[7]; 2 MAGs were found to be chimeric; and 1 did not contain phylogenetic marker genes. When correlated with biogeography, our phylogeny supports the origin of the PSC/*Phaeocystis* lineage in warm waters, with later specialization to polar waters, as suggested previously[8].

Supplementary Note 2.

Repetitive elements

Repeats: We searched the *Phaeocystis antarctica* (Pa) and *P. globosa* (Pg) genomes for interspersed repeats using REPET (Methods). We found 35% (55 Mbp) and 50% (101 Mbp) genome coverage in Pg and Pa, respectively which partially explains the higher genome size in the latter. The vast majority (55% in Pg and 50% in Pa) of the annotated repeats remained unclassified. The most abundant putative TEs are the non-autonomous TIR and TRIM/LARD elements, the latter being significantly more abundant in Pa. Elements belonging to Copia and LINE retrotransposons are the most abundant autonomous TEs. Intriguingly, homology search based on protein structures enabled the detection of a recombinase domain in ORFs from a few unclassified repeats and they were considered putative mobile elements referred to as "PutMobRec" (Supplementary Data 2).

Endogenous viruses: *Phaeocystis* sp. in known to be the host of several nucleo-cytoplasmic large DNA viruses (NCLDVs, e.g., Phaeocystis globosa virus, PgV), including Mesomimiviridae clade and their co-infection with the virophage-like elements (e.g., Polinton-like viruses) has been reported[9–11]. With its peculiar major capsid protein (MCP), the PgV virophage (PgVV) sequence subsequently turned out to become the founder of the recently proposed Polinton-like viruses (PLVs)

group[10]. Endogenous viruses can reveal ancient or cryptic viral-host interactions[12] and endogenous PLVscan protect their eukaryotic host by limiting the multiplication of their NCLDV host at the population scale. Therefore, we have searched the Pa and Pg genomes for genetic footprints of interactions with virophages/PLVs and NCLDVs.

We first found 6 and 8 loci in Pa and Pg, respectively, containing hallmark genes of virophages, polintons and PLVs and preliminary sequence comparison suggested that they represent two groups that most resemble PLVs, hereafter referred to as Phaeocystis endogenous PLV (PePLV) 1 and 2, respectively. PePLV2 copies are present only in Pa and appear to be heavily truncated (data not shown). Instead, we detected several potentially complete copies of PePLV1 as indicated by the presence of terminal inverted repeats (TIRs) at the edge of sequence conservation in a multicopy alignment (7 in Pg, 2 in Pa, ranging 20-27 kb). The PePLV1 copies have relatively low G+C content (ranging 40-48%) compared to the host genomes (65-67%) and appear as large GC-poor islands in the Pa and Pg contigs (Supplementary Fig. 8c).

One of the PLV hallmarks is a conserved MCP. Phylogenetic analysis of the MCP protein confirmed that PePLV 1 and 2 belong to the PLV and positioned the two groups as nested within the PgVV clade of PLV. None is sister to the PgVV MCP, the PePLV 1 and 2 proteins being found closest to homologs from Pleurochrysis PLV and Chrysochromulina parva virophage, respectively (Supplementary Fig. 8b). The latter also corresponds to a PLV despite its name[13]. Remarkably, the different PLVs found to associate with the haptophytes *Chrysochromulina* sp., *Pleurochysis* sp. and *Phaeocystis* sp. clade together as a subgroup within the PgVV clade.

Intriguingly, multiple sequence alignment of the PePLV1 copies presenting TIRs revealed obvious swaps in the pairs of copies sharing highest similarity among the seven Pg copies (Supplementary Fig. 8a). We assessed the presence of signals of recombination between copies using different tests implemented in RDP4[14] and we detected several events with high confidence whatever the copy assigned as parental (Supplementary Fig. 8d). We also found evidence of substantial structural variations among these copies that translate in different ORF contents (Supplementary Fig. 8e). The core PLV genes are well conserved among the Pg and Pa copies: all contain minor capsid protein (mCP) and MCP and all but one also present the packaging ATPase. Most or all copies also encode other proteins found in other PLVs including DNA primase/helicase, tyrosine recombinase and DNA cytosine methyltransferase. Several other ORFs are distributed across fewer copies or even a single copy. Many have a functional annotation including Hydrolase-lipase, tRNA methyltransferase as well as an arsenal of DNA editing enzymes comprising DNA adenine methylase, putative GIY-YIG endonuclease (also found in PgVV), endonuclease VII and intron-associated endonuclease. In

addition, we detected evidence of gene replacement with the presence of 2 types of Hydrolase/lipase proteins (inferred from structural annotation) and 2 types of DNA primases, one having best bit against virophage and PLV orthologs whereas the other has best hit against giant virus orthologs.

We also searched the Pg and Pa genomes for potential copies of endogenous NCLDV and found one locus in Pg and four loci in Pa with elevated density of NCLDV marker genes over at least 25kb. Over the region in Pg, ViralRecall[15] predicted a putative NCLDV locus spanning 47.4 kb and ~62% G+C, which is close to host genome average (65-67%). This locus was named Pg endogenous NCLDV (PgeNCLDV). The four regions in Pa share high sequence identity and their extension followed by multiple sequence alignment allowed identifying the boundaries of sequence conservation which was used to determine the extremities of each locus, one of them being truncated (Supplementary Fig. 8a). The four copies present good overall alignment with few structural variations. The copies found on scaffolds 26 and 21 catch most of the sequence diversity across copies, hereafter referred to as PaeNCLDV type 1&2, and spanning ~47.6 and ~53.6 kb, respectively. The GC content of these loci, ranging 55-56%, is in strong contrast with the relatively GC-rich flanking DNA (Supplementary Fig. 8c). We compared PgeNCLDV to PaeNCLDV loci and found only trace sequence similarity suggesting they represent independent integration of distinct viruses in Pa and Pg (data not shown).

We predicted 46 and 56 ORFs from the representative potential NCLDV loci PgeNCLDV and PaeNCLDV type 1, respectively. When compared to GenBank nr using BLASTP, we found that, out of the proteins with a hit, most (8/20 and 22/35 in PgeNCLDV and PaeNCLDV type 1, respectively) had a best hit against proteins predicted from the genomes of three Pleurochrysis sp. endemic virus isolates (1a, 1b and 2, partial genomes spanning 25-36 kb). Functional annotation allowed detecting only four out of the six core NCLDV proteins across the Pa and Pg loci: major capsid protein (MCP), packaging ATPase, VTLF3 (in Pa only) and D5-like primase-helicase while we could not detect similarities with the other core NCLDV functions DNA polymerase B and TFIIS. The Pa and Pg were also found to encode proteins with functions including Holliday Junction Resolvase and YqaJ-like viral recombinase, among others (Supplementary Data 9).

Phylogenetic analysis of the MCP protein shows that those from PaeNCLDV and PgeNCLDV are most closely related to homologs from Pleurochrysis sp. endemic viruses and form a clade together with the recently described Yaravirus isolated in *A. castellanii*[16] (Supplementary Fig. 8b). It appears that this clade of NCLDV we refer to as Yaravirus-like share relatively small genome size (the largest being PaeNCLDV type 2 with 53.6 kb) and lack some hitherto considered "core" proteins among the NCLDV. While tRNA genes were reported in the Yaravirus, none could be found in the

Phaeocystis loci or Pleurochrysis sp. endemic viruses using ARAGORN[17]. Together, this data suggests that *Phaeocystis* spp. could be infected by members from two distincts clades of NCLDV: Yaravirus-like and Mimiviridae-related. Because the prymnesiophyte *Pleurochrysis* sp. is also host of Yaravirus-like viruses, we hypothesize that it might be the case of many other haptophyte species.

Altogether endogenous PLV in Pa and Pg appear as dynamic features that may contribute to host defense against hypothetical NCLDVs. To investigate if these interactions are detectable in environmental samples, we analyzed PLV- and NCLDV-mapping reads (Supplementary Fig. 8f-g). While this analysis cannot reliably distinguish between the expression of endogenous and exogenous viruses, the data show some level of coincidence between PLVs and NCLDVs. Additionally, noticeable expression of meiotic genes is observed at some stations, suggesting a biological interaction between *Phaeocystis* and its viruses.

Supplementary Note 3.

Genome-assisted biogeography

We used data from four cruises to estimate the global abundance of Phaeocystales; 1) a part of The Sorcerer II Global Ocean Sampling Expedition – a brackish transect of the Bay of Oslo and the Baltic Sea[18]; 2) data from California Current Ecosystem (CCE) – CalCOFI[19]; 3) a meridional transect of the Atlantic Ocean[20]; 4) the global dataset of Tara Oceans, including the Tara Arctic samples[21].

Altogether, 0.96×10^{9} metagenomic reads mapped to the combined *Phaeocystis* assemblies, representing 0.9 % of all processed reads ($n=105.7\times10^{9}$) from 103 stations. This is in good agreement with previous works, assigning 0.25-3.72% of global reads, and at least 4.3% of global biomass, to *Phaeocystis* [22–25]. Specifically, according to de Vargas et al.[23], *Phaeocystis* reads have the abundance of 1,439,566, 95.2% of it from OTU 38b7 which branches in the globosa cluster. The total number of sequenced V9 data is 525-570M reads, making *Phaeocystis* abundance ~0.25 %. In Vogt et al.[22] and MAREDAT, lower estimates are ~4.3% of global biomass, which could be an overestimate, as they focus on coastal areas and biomass might not correlate linearly with rDNA due to the large DOC production of the colonies. Carradec et al.[24] report 3.72/2.5% metaG/metaT occurrence for Haptophyceae unigenes, large part of which are Phaeocystales – 1.56/0.66%, which is about twice as abundant as Isochrysidales – 0.76/0.32. In Sow et al.[25], *Phaeocystis* spp. represented ~2% of all eukaryote 18S sequences recovered.

Our data represent an underestimate because the species resolution of the mapping process is quite high, with error rate below average genetic distances (1.07 % and 1.02 % for metaG and metaT, respectively) and only ~4.1 % of reads mapping non-specifically to ungrouped assemblies

(multi-mapping). Specifically, 39.2 million reads were multi-mapping, i.e., mapped with equal probability to assemblies from different species. The average and maximum error rates of read mapping (expressed as the ratio of mismatched bases) were below the inferred average genetic distances, altogether suggesting a sufficient specificity of the mapping procedure (not shown). Within the *P. globosa* species cluster, multi-mapping reads constituted a larger fraction (20.24 %). The average read mapping error rates were comparable to the average genetic distances of the assemblies, and hence the resolution of our procedure could be affected (i.e., environmental reads could be assigned to an incorrect *P. globosa* assembly). Increasing error rate stringency would fine-tune read recruitment from environmental samples at the cost of *P. globosa* abundance underestimation, however, grouping of the assemblies into genotypes 1-3 seems to largely overcome the mapping error rate versus genetic distance problem. More data from closely related and uncultured lineages would likely increase our estimated global abundance by covering more *Phaeocystis* sequence diversity. Notably, plastid genomes recruited substantially more metaT reads than metaG reads, consistent with high photosynthetic plastid transcription (Supplementary Fig. 2) and underlining the importance of transcriptomic data in these comparisons. Since organellar transcripts are not typically analyzed in metaT studies based on polyA-enriched libraries, we also tested if the organelle-mapping reads correlate in NCOG data, where we have complementary polyA and ribosomal RNA-depleted data. For whole genome-mapping read abundance, we saw a significant correlation, with higher abundance recorded for ribo-depleted samples (Spearman's *rho* for mitochondrial data ranging 0.71-0.79, except the least abundant *P. rex* with 0.51, and *p*-values always $<1^{-20}$; Spearman's *rho* for plastid data ranging 0.78-0.82 and *p*-values always $<1^{-60}$; polar genomes were omitted from the analysis). For gene-level mapping abundances, we analyzed *P. globosa* genotype 3 and *P. cordata*. Similarly, most genes were significantly correlated (31 of 38 mitochondrial genes with *p*-value $<1^{-4}$, mean *rho* 0.42; 200 of 232 plastid genes with *p*-value $<1^{-4}$, mean *rho* 0.47).

To assess whether any of the assemblies represent polar specialists, we tested for enrichment of normalized reads from the Southern Ocean (latitude <50 °S) or the Arctic Ocean (latitude >60 °N) relative to non-polar stations (temperate + tropical). Indeed, *P. antarctica* was >500× more abundant in the Southern Ocean (i.e., TARA_SOC_28_MAG_00057: 550×; Phaant1: 567×; Antarctic multimapping reads: 708×), and *P.* cf. *pouchetii* was >140× more abundant in the Arctic than in non-polar stations. Additionally, we identified several MAGs significantly associated with polar stations (two-sided Mann–Whitney U test), namely:

- TARA_AOS_82_MAG_00183, labeled as *Phaeocystis* sp. 1, branching with *P. antarctica* and *P.* cf. *pouchetii*; 23.7× more abundant in the Arctic;
- TARA_SOC_28_MAG_00067 and TARA_SOC_28_MAG_00074, labeled as *Phaeocystis* sp. 2 and 3, respectively, branching outside the *P. globosa*/*antarctica*/*pouchetii* clade, 78.8× and >1300× more abundant in the Southern Ocean, respectively;
- TARA_SOC_28_MAG_00056 and TARA_ARC_28_MAG_00248, branching in the broader *P. jahnii* clade (within „Phaeo2“), 360× and >77.6× more abundant in the Southern and Arctic Ocean, respectively. Their branching on the timetree suggests that the former speciated ~33.3 Mya, coinciding with the first glaciation of Antarctica 34 Mya[26], while the latter speciated ~11.8 Mya, largely coinciding with its reglaciation 14 Mya[27] (for interactive tree, see: https://itol.embl.de/tree/97124473413554174171795 4)

These results suggest that *Phaeocystis* spp. have colonized polar waters multiple times through convergent evolution, although more complete genomes are needed to confirm their identity and assess their specific polar adaptations. Notably, given that *P. jahnii* is widely recognized as a mid-latitude species, the discovery of polar specialists closely related to it is unexpected and raises questions about potential competition with *P. antarctica* and *P. pouchetii*. This underscores the challenges in classifying *Phaeocystis* spp. across diverse environments, owing to their inconspicuous appearance as nanoflagellates, which likely hampers their detection and study in natural ecosystems.Among stations that recorded high *Phaeocystis* abundances (RPM>10^4, n=12), reads from mesoplankton- and microplankton-scale samples were predominant only in three. Therefore, single-cell *Phaeocystis* appear quite abundant well before colony formation is triggered. We identified the most probable drivers of *Phaeocystis* abundance and transcription using the Generalized Additive Model (GAM). We compared abundances based on metaG data with metaT data, and generally saw similar trends, although more pronounced in metaT space. Further, we elaborate on the latter results that reflect not only abundance but also physiological responses.

Pico- and nano-sized (small) fraction read abundances often positively correlated with ammonium, but not with nitrate. *Phaeocystis* abundances also often negatively correlated with diatom abundances (Supplementary Fig. 3a,e). Detectable microplankton- and mesoplankton-scale data were only available for a few stations, and while correlations with nitrate and temperature are biologically relevant, these data were not interpreted.

In small fractions, temperature was a factor that strongly distinguished the three genotypes of *P. globosa*, with genotype 1 generally more abundant at higher temperatures, genotype 2 showing peak abundance at ~17 °C, and genotype 3 showing peak abundance at ~10 °C. Genotype 3 also

appeared positively responsive to ammonium at higher concentrations. We also observed a positive relationship with chlorophyll *a* in *P. globosa* genotype 3, suggesting this genotype could occur more frequently in communities with higher algae abundances, compared with the other two genotypes that did not exhibit such correlation (Supplementary Fig. 3b). For polar species *P. antarctica* and *P. pouchetii*, nitrate, iron, and temperature were strong determinants of their abundance. Specifically, *P. antarctica* positively correlated with nitrate and had a peak abundance at lower iron levels, whereas *P. pouchetii* did not correlate with nitrate or iron, and negatively correlated with diatom abundance (Supplementary Fig. 3c,e). The abundances of other *Phaeocystis* seemed to be positively correlated with temperature and negatively correlated with nutrients and diatoms, although these trends were not always significant (Supplementary Fig. 3d-e).

Supplementary Note 4.

Functional annotation

Transporter genes: We identified 1385, 1092, and 1554 transporter genes in Phaant1, Phaglo1, and Phacord1, respectively. Most of these genes encode transporters for a broad spectrum of substrates (ABC, DMT, MFS, VIC; Supplementary Data 7). Among haptophytes, *Phaeocystis* spp. are not significantly enriched in any transporter substrate class, although Phacord1 has a higher number of Class IC and ID inorganic compound transporters as well as Class II (carbohydrate/sugar; ABC, MFS and TRAP-T families), III (amino acid/protein; ABC and AAAP families), V (vitamin/cofactor), and VI (antimicrobial/lipid) transporters than Phaant1 or Phaglo1. Uniquely among haptophytes, Phaeocystales encode a higher number of GIC transporters (amino acid or glutamate-gated ion channels), BCCT and bestrophins (putative ion channel or glycine-betaine transporters), which might point to acquisition of organic nitrogen compounds when nitrate is low. Some of these transporters were expressed in metaT data (Supplementary Data 7).

We calculated the heterotrophy index (H-ind) of Haptophyceae based on their reference genome annotations and a recently published algorithm[28] and found that Phaeocystales were comparable to other haptophytes (H-ind -26.4, -21.5, and -15.9 for Phaant1, Phaglo1, and Phacord1, respectively, compared to other haptophytes *Chrysochromulina*, *Emiliania*, *Dicrateria*, and Pavlovales sp. CCMP2436 ranging from -41.7 to -9.8). With negative H-ind values (typically between -400 and -100) associated with obligate phototrophs and positive values (between 0 and 200) associated with heterotrophy, we corroborate that some lineages of Haptophyceae might be mixotrophic in specific conditions[29] (also Supplementary Fig. 5c,d). Interestingly, while *P. globosa* and *P. antarctica* have been found capable of growing mixotrophically based on an increased presence of phagocytosis

markers[29], *P. cordata* lacks these markers, but is known to form symbiotic relationships with larger planktonic eukaryotes from the clade Acantharea (Rhizaria)[30,31].

Genome-scale metabolic models: To further test if the metabolism of *Phaeocystis* supports a mixotrophic mode, we generated genome-scale metabolic models (GEMs) for the three reference *Phaeocystis* genomes. Our reconstruction process leveraged previously established GEMs of other microalgae, including *Cylindrotheca closterium*[32], *Picochlorum renovo* (Tec-Campos, Tibocha-Bonilla, Passi, Canto-Encalada, Dahlin, Rosenbach, Bray, Guarnieri, Martinez, Butler, Early, Zuñiga, and Zengler; unpublished), and *Chlorella vulgaris*[33]. Phaglo1 and Phaant1 share more metabolic features, whereas Phacord1 displayed more unique metabolic reactions and metabolites (Supplementary Fig. 6a). To further investigate these differences, we conducted simulations of these GEMs under photoautotrophic and mixotrophic conditions. Phototrophic environment assumed specific inputs, including uptake fluxes of CO_2 and light, whereas mixotrophic environments additionally assumed uptake fluxes of organic carbon sources and one of three major nitrogen sources (nitrate, ammonium, or urea). First, we estimated overall growth and the distribution of metabolic fluxes across various cellular compartments. Interestingly, Phacord1 would not grow mixotrophically on glucose (not shown) but can grow on acetate as a carbon source (Supplementary Fig. 6b). Regarding nitrogen sources, nitrate elicited different flux changes than urea and ammonium (Supplementary Fig. 6b). Phaant1 showed a notably higher mitochondria-to-plastid flux ratio (M/P ratio) compared to Phaglo1 or Phacord1 in phototrophic conditions, more prominent under mixotrophy with nitrate (Supplementary Fig. 6b), in line with generally higher mitochondria-to-plastid transcription rates *in situ* (Fig. 2c). Supply of organic nitrogen led to increased flux through the plastid in Phaant1, and thus in a diminished M/P ratio, whereas Phaglo1 and Phacord1 responded with an increase in the mitochondrial flux and increased M/P ratio (Supplementary Fig. 6b).

During phototrophic growth, most metabolic flux, modeled as $mmol.gDW^{-1}.h^{-1}$, passed through energy metabolism and transport, followed by glycolysis. Phaant1 and Phaglo1 exhibited a rather small flux via the urea cycle, whereas Phacord1 showed a moderate flux through this important nitrogen hub (Supplementary Fig. 6c). In mixotrophic conditions, various nitrogen sources differently affected metabolic fluxes; in all *Phaeocystis* GEMs, nitrate increased the flux through the nitrogen metabolism, ammonium decreased the flux through the energy metabolism, and urea decreased the flux through the urea cycle (Supplementary Fig. 6c). These differences clearly reflect where each nitrogen form becomes integrated into the central metabolism. Yet, *Phaeocystis* GEMs also showed species-specific responses, with Phaant1 and Phaglo1 being more alike compared to Phacord1. For instance, urea led to increased flux through the nitrogen metabolism and decreased

flux through glycolysis in Phaant1 and Phaglo1, but in Phacord1 urea increased flux through transport and core carbon metabolism from glycolysis to the TCA cycle and lipid metabolism, with a decreased flux through the pentose-phosphate cycle (Supplementary Fig. 6c). Ammonium led to decreased flux through glycolysis and increased flux through the nucleotide metabolism and the urea cycle in Phaant1 and Phaglo1, with a Phaant1-specific increase of the flux through cofactor metabolism (consistent with the increased flux through the plastid). In contrast, in Phacord1, ammonium led to an increased flux through transport and lipid metabolism (Supplementary Fig. 6c). The mixotrophic capabilities of *Phaeocystis* are therefore likely dependent on specific sources of both carbon and nitrogen. Importantly, *Phaeocystis* spp. seem to utilize a urea cycle to redistribute cellular nitrogen, with carbamoyl-phosphate synthesis localized both in the mitochondria and the cytosol, an arrangement homologous to that of diatoms and *Emiliania*[34] (phylogenetic trees in OSF repository https://osf.io/vka93/). In summary, our *Phaeocystis* GEMs suggest variations in metabolic arrangements that might be driven by both environmental adaptation (e.g., the effect of organic nitrogen to M/P ratio) and evolutionary distance (e.g., fluxes through nitrogen metabolism).

Supplementary Note 5.

Physiological responses of Phaeocystales in polar and temperate oceans

To investigate nutrient-related transcriptional responses in contrasting oceanic regions, we compared Pfam expression in the Southern Ocean (CICLOPS project), California Current Ecosystem (CCE; CalCOFI/NCOG project), and Arctic Ocean biotopes (pole-to-pole and Tara Oceans subsets; Supplementary Data 3). We had a particular interest in Southern Ocean responses, perhaps adaptations, since annual blooms of *Phaeocystis* in this iron-limited biotope[35] have a global primary productivity impact.

We assembled the metatranscriptomes, extracted all ORFs with taxonomic affiliation to *Phaeocystis* spp., grouped them by MCL clustering, and annotated and quantified the resulting clusters across euphotic samples (Methods). We obtained 660,168 ORFs grouped into 69,485 orthogroups (including 4,175 singletons), of which 7,339 could be assigned Pfam annotations. After filtering to ≦10 in at least two biotopes, in total 7,316 orthogroups remained (2,772 with Pfam annotations), which were further grouped to 2,466 Pfams.

First, we identified orthogroups with the highest average expression (top 1000) and determined which corresponding Pfams occurred uniquely for each biotope. In the Southern Ocean data, 91 Pfams occurred uniquely, notably including iron-responsive Pfams (PF06799 iron-responsive protein, PF02535 putative heavy metal-binding protein/transporter, PF01126 heme

oxygenase, and PF02678 putative Fe^{2+}-containing nuclear regulator pirin) and a B_{12}-independent methionine synthase (PF01717)[36], suggesting iron-B_{12} co-limitation (Supplementary Data 6). These unique Pfams also participate in functions typically associated with *P. antarctica*, i.e. sulfur metabolism (PF00916 sulfate permease) and ice association (PF11999 ice binding protein). Pfams highly expressed in both the Southern Ocean and the Arctic (n=62) include photosynthesis-related proteins and oxidative stress-related redoxins (PF00301 rubredoxin, PF00462 glutaredoxin). In the Arctic data, unique highly expressed Pfams (n=94) included additional photosynthesis-related proteins, stress-related multi-copper oxidases (PF07731/PF07732) and Fe/Mn superoxide dismutates (PF00081/PF02777), and enzymes of the polyamine biosynthesis (PF01536 adenosylmethionine decarboxylase and PF01564 spermine/spermidine synthase), which might partake in nitrogen storage and/or osmoprotection. In the temperate CCE data, unique highly expressed Pfams (n=296) included proteins associated with intracellular transport (coatomer subunits and Sec23/Sec24 protein), tetrapyrrole biosynthesis, and central carbon metabolism (glycolysis, TCA cycle) (Supplementary Data 6).

Next, we tested which orthogroups and Pfams are differentially enriched between the three biotopes. Due to the compositional nature of the expression data and the use of TPM normalization (Methods 4), the results of these Kruskal-Wallis tests should be interpreted as differential enrichment rather than absolute differential expression. CCE had the most differentially expressed orthogroups and Pfams (1527 and 834, respectively), whereas the Southern Ocean had the fewest (262 orthogroups and 134 Pfams); 566 orthogroups and 368 Pfams showed differential expression between all three datasets (main text Fig. 3). In both polar biotopes, significantly increased Pfams are associated with cytochrome c oxidase and photosystem II biogenesis, electron transfer chains, oxidative stress mitigation (rubredoxin, glutaredoxin, peroxidase), and metal homeostasis (PF01491 frataxin, PF04145 Ctr high-affinity copper transporter, PF02659 Mn^{2+} efflux pump, PF03203 Hg^{2+} resistance protein) (Supplementary Data 6, main text Fig. 3d). Additional significantly increased Pfams belong to one carbon pool metabolism and lysine/RNA/phospholipid methyltransferases, and multiple domains of unknown function, raising questions about their roles in polar marine environments. In Southern Ocean specifically, significantly increased Pfams (i.e. Pfams in significance groups "all-to-all", "Southern", and "other", Fig. 3, with >50% proportional TPM in CICLOPS data) included sulfate metabolism (including organosulfur compound production, Fig. 3f, Supplementary Fig. 2b), metal homeostasis (PF06799, PF02535, PF01126, PF02678, mentioned above, and PF01906 heavy metal binding protein, PF05023 phytochelatin synthase, Fig. 3d), and B_{12}-independent methionine synthesis (via metE), thus corroborating the top 1000 orthogroup results regarding iron-

B_{12} co-limitation (Supplementary Data 6). Although Phaeocystales ISIP2a expression is not significantly higher than in CCE, the complement of other iron-interacting proteins and a flavodoxin-enriched photosynthetic apparatus might alleviate iron-deficiency stress sufficiently. A strong reliance on B_{12}-independent metE seems to be a feature not only limited to the CICLOPS sampling sites, but also other areas of the Southern and Arctic Oceans (Fig. 3; Arctic vs SOC: $p = 1.09e-07$ (BH corrected: 1.09e-07), Vargha-Delaney A = 0.834, median difference 0.51 [95% CI: 0.26, 0.64]; Arctic vs CCE: $p = 9.76e-11$ (BH corrected: 1.46e-10), Vargha-Delaney A = 0.249, median difference -0.17 [95% CI: -0.41, -0.07]; SOC vs CCE: $p = 1.02e-16$ (BH corrected: 3.07e-16), Vargha-Delaney A = 0.033, median difference -0.69 [95% CI: -0.75, -0.60]). Generally, these patterns are supported by ANCOM-BC2, which estimates absolute differential expression (Supplementary Data 6). However, the estimated CCE biomass is also consistently lower than in the polar biotopes (average Phaeocystales CCE expression is estimated to be 35.7 and 37.3 % that of Southern Ocean and Arctic, respectively).

In CCE, similarly to unique Pfams, significantly increased Pfams (i.e. Pfams in significance groups "all-to-all", "temperate", and "other", Fig. 3, with >50% proportional TPM in NCOG data) included functions associated with photosynthesis (tetrapyrrole biosynthesis, non-mevalonate terpenoid synthesis, electron transfer chain proteins, and central carbon metabolism) and intracellular transport (coatomer, Sec23/Sec24, vacuolar protein sorting). Interestingly, ammonium transporter, multiple peptidases, and PF00530 Scavenger receptor cysteine-rich domains were found significantly increased as well (Supplementary Data 6, Fig. 3d), suggesting nitrogen limitation and increased endocytosis (Supplementary Fig. 6). These results are only partly supported by ANCOM-BC2 (Supplementary Data 6), possibly because of the overall smaller Phaeocystales biomass in CCE. Specifically, of the above enriched Pfams, only some were shown differentially overexpressed in CCE relative to polar data by ANCOM-BC2 (trypsin PF00089, peptidase M48 PF01435, scavenger receptor cysteine-rich domain PF00530, RuBisCO small chain PF00101, ATP synthase subunit A, photosystem protein PF00124, cytochrome F PF01333).

To correlate Pfam abundance with environmental parameters, we used the Tara Oceans Gene Atlas MATOU v2 and the associated metadata[37] (Methods). We found 625,346 unigenes annotated as Phaeocystaceae, corresponding to 4,548 Pfam annotations present in the metaT occurrence tables, of which 3,150 passed the mean occurrence $>10^{-5}$ threshold, and 1,038 were found significantly correlated with predicted total iron levels ("Iron_5m"; Benjamini-Hochberg-corrected two-sided Spearman's *rho* at p-adj<0.01 level; Supplementary Data 6). Positively correlated Pfams, corresponding to iron-replete response, included ammonium and nitrite transporters (PF00909 and PF01226), sulfur transporters (PF01925 and PF04143), a periplasmic copper-binding protein

(PF05048), and ubiquitin-dependent proteolysis and autophagy-associated Pfams. Negatively correlated Pfams, corresponding to iron-starvation response, not only induced multiple iron-and copper-responsive Pfams (PF06799 iron-responsive protein, PF02535 putative heavy metal-binding protein/transporter, PF07692 low iron-inducible protein ISIP2a, PF04145 Ctr), but also diverse anabolic pathways (shikimate, terpenoid, and tetrapyrrole biosynthesis, sulfotransferases, and central carbon metabolism), along with nitrogen recycling (peptidases, urea transporter). Iron starvation also triggers oxidative stress response (tocopherol and lycopene cyclases, glutathione peroxidase, rubredoxin) and strongly correlates with flavodoxin expression and flavin synthesis (PF00925 GTP cyclohydrolase II, PF00926 3,4-dihydroxy-2-butanone 4-phosphate synthase; Supplementary Data 6), consistent with previous reports[38,39]. This iron-limitation responsive expression is mirrored by specific gene family expansions in the Southern Ocean (Fig. 3i; Benjamini-Hochberg-corrected Mann-Whitney U test (DOF), Vargha-Delaney effect size A:
$U_{NiR}(143)=2811.0$, $p=2.83e\text{-}06$, p-adj=1.41e-05, A=0.761, median copies 1.90 [95% CI 0.24-2.69];
$U_{dsyB}(145)=2436.0$, $p=0.015$, p-adj=0.017, A=0.621, copies 0.94 [95% CI 0.71-1.01];
$U_{ISIP2a}(145)=2559.0$, $p=0.0032$, p-adj=0.0041, A=0.653, copies 1.46 [95% CI 1.12-1.71];
$U_{ISIP3}(145)=2839.0$, $p=3.23e\text{-}05$, p-adj=6.46e-05, A=0.724, copies 1.08 [95% CI -0.49-1.68];
$U_{VIT}(143)=2566.0$, $p=3.58e\text{-}04$, p-adj=5.96e-04, A=0.694, copies 1.35 [95% CI 0.93-1.42];
$U_{CA}(143)=2526.0$, $p=6.99e\text{-}04$, p-adj=9.99e-04, A=0.683, copies 0.69 [95% CI 0.07-1.09];
$U_{Alma1}(145)=2875.0$, $p=1.6e\text{-}05$, p-adj=3.99e-05, A=0.733, copies 1.82 [95% CI 1.27-2.38];
$U_{Fd}(145)=2090.0$, $p=0.28$, p-adj=0.28, A=0.533, copies 1.37 [95% CI 1.13-1.51];
$U_{Fld}(145)=3301.0$, $p=5.42e\text{-}10$, p-adj=5.42e-09, A=0.842, copies 2.09 [95% CI 1.53-2.27];
$U_{xr}(145)=2879.0$, $p=1.48e\text{-}05$, p-adj=3.99e-05, A=0.734, copies 1.37 [95% CI 0.70-2.25]).

Our results suggest there are significant regional transcriptomic trends in the temperate CCE and the polar biotopes of the Arctic and the Southern Ocean that are specifically adjusted by Phaeocystaceae to respond to local environmental conditions, strongly driven by nutrient availability.

Supplementary Note 6.

Gene family expansion and horizontal gene transfer

In haptophytes, significantly expanded Pfams belong to most major biological processes, suggesting genome evolution in this lineage generally relies on gene duplication and novelty. Haptophyte genomes indeed encode large numbers of genes (Fig. 4); considering orthogroups present in at least 50% of prymnesiophytes as core, a “core haptophyte genome” would be 9,121

orthogroups and 16,516 genes. Yet, there do not seem to be strong indications of whole genome duplication in haptophytes. The average size of a core haptophyte orthogroup in each species is ~1.6-1.8 (depending on whether Pavlovales or genomic data only are included), but the median equals 1 for ~77% of them. The more likely explanation is frequent duplication facilitated by intergenic simple repeat recombination. The highest Pfam enrichment we found in PSC, Emihu1, and Phacord1, which also have the most gene-rich genomes in our comparison, and which underwent an expansion of 1,846, 2,064, and 1,322 Pfams, respectively (Supplementary Data 8). Among these, hundreds of genes constituted the significantly expanded Pfams (639 genes in 59 Pfams in PSC, 1,558 genes in 119 Pfams in *E. huxleyi*, and 1,261 genes in 166 Pfams in Phacord1). The number of contracted Pfams was comparable, 1,995 in PSC (13 significant), 2,082 in Emihu1 (39 significant) and 598 in Phacord1 (7 significant), suggesting a shift in gene family composition. With the incomplete nature of the PSC dataset in this analysis, compiled by combining four closely related MAGs and removing redundancy on 95%-level sequence identity, it is difficult to infer true gene family expansions and contractions. Nevertheless, our results suggest that the functional composition of the PSC genomes is quite like that of *Phaeocystis* spp. with a few expansions of regulators of chromatin condensation, G-protein and phosphatidylinositol signaling, and serine proteases. The content of sulfotransferases and extracellular structural proteins is generally not statistically different from colony-forming species.

Phaeocystis spp., represented here by Phaant1, Phaglo1, and Phacord1, showed statistically significant Pfam expansions that partly underlie their specific biology. The first group of expanded families probably relate to the formation of extracellular structures (such as scales and star-shaped filaments), consisting of glycoside transferases, sugar phosphate transporters, chitin-binding domains, type III fibronectins, and exostosins (Supplementary Data 8, ref[1]). Specific expansions were also seen in photosynthesis and redox relay (rhodopsins IPR001425, notably including virus-derived heliorhodopsins, iron-responsive xanthorhodopsins, and haptophyte anion channelrhodopsins, see phylogenetic trees in OSF repository; NADP-binding and glutaredoxin domains). Furthermore, sulfotransferase (IPR000863, IPR005331, IPR018011, IPR037359, and IPR040632) domains were substantially enriched in *Phaeocystis* spp.; these are candidates potentially involved in the extensive metabolism of organosulfur biomolecules, including extracellular sulfated glycans. Among transporters, bestrophins (IPR021134), inorganic ion channels, type I and II ABC transporters, and major facilitator superfamily domains were abundantly present, suggesting the spectrum of transported metabolites in *Phaeocystis* is quite broad (Supplementary Note 4).

How biological processes are regulated in *Phaeocystis* has not been experimentally addressed, but post-translational regulation could play a major role[40]. Consistent with this view,

many protein-modification and signal-transduction Pfams were found enriched (Fig. 4, Supplementary Data 8). Most frequent protein-modification domains include FKBP-type peptidyl-prolyl isomerase (IPR001179), ubiquitin-like (IPR000626), and lysine methyltransferase (IPR019410). Among the most abundant Pfams are also ShKT domains. While the function of these small six-cysteine domains is not clear, in many sequences they co-occur with protein-modification domains, such as proline 4-hydroxylase and serine α-galactosidase, or secondary metabolism tyrosinase. The presence of signal peptides in ShKT-domain proteins suggests a localization via secretory pathways, and iron-dependent expression patterns corroborate their ecological importance[41]. In Phacord1, signal transduction Toll-like kinases (IPR000157), and calcineurin-like phosphatases (IPR004843) are significantly expanded compared to other *Phaeocystis*, whereas in Phaant1, specific expansions in cyclins (IPR039361/IPR004367/IPR006671), chemical-gated ion-channels (IPR006201), and small GTPases (IPR001806) could contribute to signal transduction complexity. From transcription factors, only zinc-finger domains seem enriched in all three genomes. In Phaglo1, a C-5 cytosine methyltransferase family (IPR001525; enriched by 24 genes) specifically expanded, likely involved in heterochromatin maintenance and modulation of gene expression. Some cold-shock proteins are lost from Phaant1, and Phacord1 is specifically enriched in SNF2-like and RCC2 chromatin regulators (IPR000330, IPR000408), helicase-associated domains (IPR005114), and poly-A polymerase domains (IPR012317), which suggests somewhat diversified regulation of expression between *Phaeocystis* spp.

Phacord1 harbors expanded families from most euKaryotic Orthologous Groups (KOG) functional classes (Supplementary Data 8). Several processes of nutrient redistribution were found particularly expanded, including lipid, nitrogen, and sulfur metabolism. Rearrangements in nitrogen metabolism are indicated by the expansion of amine oxidase, amino-acid specific transporters (glutamate receptor IPR001320), and formamidase domains. Six peptidase families are also expanded in Phacord1, although their involvement in protein turnover or perhaps nitrogen redistribution is not clear. Expansions in taurine dioxygenase (TauD) and sulfate transporter (SulP) families suggest additional adjustments in sulfur metabolism compared to other *Phaeocystis*.

With more contractions, fewer expansions, and fewer Pfams detected overall (4,842 in Phaglo1, 5,064 in Phaant1, 5,652 in Phacord1), Phaglo1 showed lower domain richness than other *Phaeocystis* or PSC (main text Fig. 4b, Supplementary Data 8). Families significantly enriched in Phaglo1 include Pfams with extracellular functions (von Willebrand and C-type lectin) and ammonium transporters. In *P. antarctica*, von Willebrand proteins were shown to be iron-responsive and hypothesized to participate in colonial matrix formation[41]. Therefore, similar functions are

expected for these proteins in *P. globosa* as well, although genes encoding von Willebrand domains are abundant also in non-colony forming Phacord1 and PSC.

Like Phacord1, and in contrast to Phaglo1, Phaant1 showed expansions in most functional classes. Genes from these expanded families do not seem to be deteriorated copies, as their average RPM is higher in Phaant1 than in Phaglo1 (main text Fig. 4b), suggesting their active transcription. As such, they likely represent adaptive portions of the Phaant1 genome, and may contribute to the ecological success of *P. antarctica* in the Southern Ocean. Notably, nitrite/sulfite reductase and carbonic anhydrase domains are significantly expanded in Phaant1, perhaps enhancing assimilation capabilities of inorganic nitrogen, sulfur, and carbon. A putative vacuolar cation transporter family, VIT, could participate in Fe^{2+} uptake and storage. In lipid metabolism, we see expansions for lipase (IPR002921) and lipid trafficking (IPR006634) domains. Three peptidases were found enriched (FtsH, S33, S54), of which FtsH is an important plastid and mitochondrial housekeeping peptidase.

A total of 183 horizontal gene transfer (HGT) events were recorded, passing our most stringent criteria (Methods 8). Most HGTs originated in stramenopiles, dinoflagellates, and opisthokonts. A similar number of events (221), where ingroup monophyly was seen, was classified as acquired ancestrally at the Haptophyta level, with a various number of haptophyte sister sequences (mean=1.73; stdev=3.81). Functionally, the HGT genes contribute to a variety of functions (Fig. 5) without a clear pattern, suggesting a largely stochastic process. Yet, three HGTs might have imparted significant new functions to *Phaeocystis* (Supplementary Data 8). A plastid-targeted thiamine thiazole synthase (THI4) was aquired by *P. antarctica* and *P. globosa*; this enzyme catalyzes a thiamine biosynthesis (vitamin B_1) reaction and has a crucial function for energy metabolism in plants and algae. Consistently, the *Phaeocystis* homologs are relatively well detectable in metaT data (mean RPM 0.44-0.74). Among haptophytes, Pavlovales use a more distantly related THI4, whereas other Prymnesiophyceae employ a different enzyme Thi6, or rely on pyrimidine precursor uptake[42]. Furthermore, *P. antarctica* acquired a NADP-dependent amino acid dehydrogenase of the Glu/Leu/Phe/Val dehydrogenase family (glutamate according to the best BLAST hit); the gene is duplicated on scaffold 11, and one of the copies appears to have a plastid-targeting presequence. The genes are well detectable in metaT data (mean RPM 0.73/0.74) and may partake in anabolic ammonium assimilation or nitrogen redistribution[43]. *P. globosa* and *P. cordata* only seem to use FAD and NAD-dependent amino acid dehydrogenases. Finally, the most straightforward adaptive novelties of *P. antarctica* are ice-binding proteins (IPR021884; 20 genes), unseen in the warmer-water algal genomes assayed here, yet common in polar algae[44,45]

(https://itol.embl.de/tree/78442411402963716372439 49). In our data, we detected ice-binding proteins with 0-0.18 mean RPM.

Supplementary Note 7.

Transcriptomic analyses of *P. globosa*

Many of these conditions were represented by single biological replicates, so the significance of differential expression was assessed by Analysis of Sequence Counts (ASC)[46], employed in previous works with similar experimental designs[40,47,48].

Timelapse of exogenous PgV infection: PgV-07T triggered a distinctive response, as determined by a posterior-probability differential expression algorithm ASC[46]. At 4 hours post infection [T4; 79 increased, 156 decreased KEGG orthologs (KOs); twofold change, posterior probability (post-*p*) > 0.95], relatively few metabolic pathways were affected, while ribosomal proteins were consistently and significantly increased (Supplementary Data 10). Spliceosome components and proteins involved in Golgi-directed secretory pathways and endocytosis were significantly decreased, perhaps hindering the host's capacity to express nuclear-encoded genes. At 24 hours post infection (T24, 50 increased, 987 decreased KOs), most metabolic pathways were significantly decreased, along with processes of protein expression and translocation, including ribosomal proteins. This suggests that the viral infection caused a halt in host biological processes by T24 (Supplementary Data 10), consistent with PgV's latent period of 10-16 hours[49]. The only significantly increased proteins at this point were endo- and exonucleases, DNA-repair polymerases, and calcium channels. Calcium is often associated with cytoskeletal rearrangements and motility, so the observed changes are consistent with altered intracellular trafficking. As an alternative approach, we identified co-expressed genes using UMAP clustering[50], looking for expression profiles consistent with progressive increase or decrease in virus-infected samples compared to controls. Clusters with potentially virus-responsive genes were enriched in similar biological processes as above, i.e. genes with gradually decreasing expression related to metabolism and gene expression (but not ribosomal proteins, which largely remained unclustered), whereas genes with gradually increasing expression did not seem to contain systematically enriched biological functions, except DNA polymerase. PePLV loci found in Phaglo1 seemed unaffected by the PgV infection.

Colony development: Several previous works addressed molecular changes in *P. globosa* developing colonies[51–54]. While two of the works featured controlled laboratory experiments focused on the molecular processes of early colony development (96 hrs[51] and 36-120 hrs[52] post colony induction), only some of their significant conclusions aligned. Clearly, the morphotype change to

colonial cells involves loss of motility and metabolic changes aimed at extracellular polysaccharide synthesis. Yet, the details of the process have not been clarified. Our data (96 increased, 82 decreased KOs) suggests that photosynthesis and translation are increased in colonial cells, whereas lower glycolysis, tricarboxylic acid cycle, and urea cycle are decreased (Supplementary Data 10), consistent with metabolic rearrangements and accumulation of polysaccharides.

Light transition: Major changes were observed in response to light exposure after prolonged (67 hours) darkness in nitrogen-rich conditions. These largely involved metabolic pathways (significant increase in photosynthesis including rhodopsins, amino acid synthesis, fatty acid desaturation, central carbon metabolism including amino- and nucleotide-sugar synthesis), molecular as well as cellular processes (increased expression of the subunits of RNA polymerase, spliceosome, ribosome and aminoacyl-tRNA synthetases, ubiquitin-mediated and proteasomal proteolysis; increased endocytosis and vesicle recycling, secretory pathway via Golgi, possibly plastid targeting via ERAD/SELMA; decreases in dynein and kinesin motors that support transport along microtubules) (Supplementary Data 10). These results suggest a major transition from dark metabolism to photosynthesis, supposedly affecting flagellar motility through decreased dynein/kinesin-dependent transport.

Nitrate supplementation: Nitrate-amended cultures (880 μM NO_3^- versus 0.37 μM NO_3^- ambient concentration) showed responses that were partially mirrored in cells entering a stationary phase 18 days post inoculation. They showed increase in anabolic reactions (gluconeogenesis, pentose-phosphate cycle, amino acid, fatty acid, chlorophyll, rhodopsin, and terpenoid biosynthesis, as well as DNA replication and translation), whereas stationary cultures showed an onset of energy-saving mechanisms in both catabolic (decrease in fatty acid oxidation and citrate cycle) and anabolic pathways (decrease in gluconeogenesis, pentose-phosphate cycle, amino acid, chlorophyll and rhodopsin synthesis, replication, and translation; increase in pyrimidine salvage) (Supplementary Data 10). Stationary cells also, interestingly, showed increased expression of protective compounds (glutathione) and fatty acid desaturases (delta-6 and delta-9), reminiscent of nitrogen starvation in *Symbiodinium* and green algae[55,56]. Meanwhile, ammonium amendment (100 μM NH_4^+) resulted in negligible changes, with only significant increase in urea cycle and DNA replication. Nitrogen compound transporters showed changes in expression across conditions, too, with highest nitrite transporter expression under exponential-growth nitrate supplementation and highest nitrate transporter expression under nitrate starvation (Supplementary Fig. 6). Nitrogen source therefore plays a major role governing cellular growth in *Phaeocystis*, and the number of significant changes compared to ammonium amendment suggests that nitrate rewires the metabolism more thoroughly,

perhaps due to being an important electron sink, similarly as in diatoms[57]. Consistently, unlike reduced-form nitrogen, nitrate is known to strongly affect colony formation in bloom-forming *Phaeocystis* spp.[58–60]. Yet, as reduced forms of nitrogen can be directly assimilated, and likely remain significant for all morphotypes, a more targeted study should address crosstalk between the uptake of various forms of nitrogen in other strains and species of *Phaeocystis*. Importantly, *Phaeocystis* have been found to employ mixotrophy via ingesting bacteria in the North Sea[29], particularly in phosphate-limited conditions, providing further evidence on the trophic versatility of these algae. The contributions of bacterial feeding, osmotrophic uptake of nutrients, and phototrophy to the overall nutrient budget under various growth conditions will need to be addressed to better understand the roles of flagellates in *Phaeocystis* life cycles.

Trace metal limitation: Iron limitation, and particularly iron-manganese co-limitation, promotes oxidative stress and a reorganization of the photosynthetic apparatus in *P. antarctica*[38]. For trace metal limitation experiments, we observed few significant changes overall (Supplementary Data 10). This may be owing to difficulties obtaining truly limiting conditions for these trace elements in out experimental setting. Notably, however, flavodoxin was significantly downregulated in iron-replete conditions, in line with other results (Fig. 3g-i, Supplementary Note 5).

Variable transcript allocation ratio (VTAR): Defined as the ratio of transcripts in the significantly increased and decreased pools and characterizing an organism's ability to transcriptionally respond to environmental cues, VTAR demonstrated that haptophytes generally do not capitalize on rapid nutrient inputs, as opposed to diatoms that act as r-selected competitors for nutrients[40]. Here, we used VTAR to test if our experimental conditions elicit major transcriptomic shifts in *P. globosa*. Our data suggest that some conditions lead to more efficient transcript reallocation than others (Supplementary Fig. 6e). In particular, the dark/light transition resulted in 4-times higher transcript allocation in the increased pool (VTAR=4.2), followed by less efficient transcript reallocation during transition to stationary cells (VTAR=1.33) and nitrate amendment (VTAR=1.18). In contrast, in cells transitioning to colonies, the increased pool consisted of fewer transcripts compared to the decreased pool (VTAR=0.03 or 0.17 depending on the annotation algorithm), suggesting that most of new transcription for this morphotype switch lies in unannotated genes, or that little new transcription is required. Similarly, PgV infection caused a decrease in new host transcription ($VTAR_{T4}$=0.42, $VTAR_{T24}$=0.16), consistent with hijacking of the host's expression machinery by the virus.

REFERENCES

1. Chen, N. *et al.* Chromosome-scale genome assembly reveals insights into the evolution and ecology of the harmful algal bloom species *Phaeocystis globosa* Scherffel. *iScience* **27**, 110575 (2024).
2. Hovde, B. T. *et al.* The mitochondrial and chloroplast genomes of the haptophyte *Chrysochromulina tobin* contain unique repeat structures and gene profiles. *BMC Genomics* **15**, 604 (2014).
3. Yang, P. *et al.* Phylogeny and genetic variations of the three genome compartments in haptophytes shed light on the rapid evolution of coccolithophores. *Gene* **887**, 147716 (2023).
4. Song, H., Chen, Y., Liu, F. & Chen, N. Large differences in the haptophyte *Phaeocystis globosa* mitochondrial genomes driven by repeat amplifications. *Front Microbiol* **12**, 676447 (2021).
5. Ondov, B. D. *et al.* Mash: fast genome and metagenome distance estimation using MinHash. *Genome Biol* **17**, 132 (2016).
6. Smith, D. R., Arrigo, K. R., Alderkamp, A. C. & Allen, A. E. Massive difference in synonymous substitution rates among mitochondrial, plastid, and nuclear genes of *Phaeocystis* algae. *Mol Phylogenet Evol* **71**, 36–40 (2014).
7. Delmont, T. O. *et al.* Functional repertoire convergence of distantly related eukaryotic plankton lineages abundant in the sunlit ocean. *Cell Genomics* **2**, 100123 (2022).
8. Medlin, L. & Zingone, A. A taxonomic review of the genus *Phaeocystis*. *Biogeochemistry* **83**, 3–18 (2007).
9. Santini, S. *et al.* Genome of Phaeocystis globosa virus PgV-16T highlights the common ancestry of the largest known DNA viruses infecting eukaryotes. *Proc Natl Acad Sci U S A* **110**, 10800–10805 (2013).
10. Krupovic, M., Bamford, D. H. & Koonin, E. V. Conservation of major and minor jelly-roll capsid proteins in Polinton (Maverick) transposons suggests that they are bona fide viruses. *Biol Direct* **9**, 6 (2014).
11. Roitman, S. *et al.* Isolation and infection cycle of a polinton-like virus virophage in an abundant marine alga. *Nat Microbiol* **8**, 332–346 (2023).
12. Blanc, G., Gallot-Lavallée, L. & Maumus, F. Provirophages in the Bigelowiella genome bear testimony to past encounters with giant viruses. *Proc Natl Acad Sci U S A* **112**, E5318–E5326 (2015).
13. Stough, J. M. A. *et al.* Genome and environmental activity of a *Chrysochromulina parva* virus and its virophages. *Front Microbiol* **10**, 703 (2019).
14. Martin, D. P., Murrell, B., Golden, M., Khoosal, A. & Muhire, B. RDP4: Detection and analysis of recombination patterns in virus genomes. *Virus Evol* **1**, vev003 (2015).
15. Aylward, F. O. & Moniruzzaman, M. ViralRecall – A flexible command-line tool for the detection of giant virus signatures in ’omic data. *Viruses* **13**, 150 (2021).
16. Boratto, P. V. M. *et al.* Yaravirus: A novel 80-nm virus infecting *Acanthamoeba castellanii*. *Proc Natl Acad Sci U S A* **117**, 16579–16586 (2020).
17. Laslett, D. & Canback, B. ARAGORN, a program to detect tRNA genes and tmRNA genes in nucleotide sequences. *Nucleic Acids Res* **32**, 11–16 (2004).

18. Zeigler Allen, L. *et al.* The Baltic Sea virome: Diversity and transcriptional activity of DNA and RNA viruses. *mSystems* **2**, e00125-16 (2017).
19. James, C. C. *et al.* Influence of nutrient supply on plankton microbiome biodiversity and distribution in a coastal upwelling region. *Nat Commun* **13**, 2448 (2022).
20. Martin, K. *et al.* The biogeographic differentiation of algal microbiomes in the upper ocean from pole to pole. *Nat Commun* **12**, 5483 (2021).
21. Salazar, G. *et al.* Gene expression changes and community turnover differentially shape the global ocean metatranscriptome. *Cell* **179**, 1068–1083 (2019).
22. Vogt, M. *et al.* Global marine plankton functional type biomass distributions: *Phaeocystis* spp. *Earth Syst Sci Data* **4**, 107–120 (2012).
23. De Vargas, C. *et al.* Eukaryotic plankton diversity in the sunlit ocean. *Science (1979)* **348**, 1261605 (2015).
24. Carradec, Q. *et al.* A global ocean atlas of eukaryotic genes. *Nat Commun* **9**, 373 (2018).
25. Sow, S. L. S., Trull, T. W. & Bodrossy, L. Oceanographic fronts shape *Phaeocystis* assemblages: A high-resolution 18S rRNA gene survey from the ice-edge to the equator of the South Pacific. *Front Microbiol* **11**, 1847 (2020).
26. Zachos, J. C., Quinn, T. M. & Salamy, K. A. High-resolution (10k years) deep-sea foraminiferal stable isotope records of the Eocene-Oligocene climate transition. *Paleoceanography* **11**, 251–266 (1996).
27. Leutert, T. J., Auderset, A., Martínez-García, A., Modestou, S. & Meckler, A. N. Coupled Southern Ocean cooling and Antarctic ice sheet expansion during the middle Miocene. *Nat Geosci* **13**, 634–639 (2020).
28. Alexander, H. *et al.* Eukaryotic genomes from a global metagenomic data set illuminate trophic modes and biogeography of ocean plankton. *mBio* **14**, e0167623 (2023).
29. Koppelle, S. *et al.* Mixotrophy in the bloom-forming genus *Phaeocystis* and other haptophytes. *Harmful Algae* **117**, 102292 (2022).
30. Decelle, J. *et al.* An original mode of symbiosis in open ocean plankton. *Proc Natl Acad Sci U S A* **109**, 18000–18005 (2012).
31. Decelle, J. *et al.* Algal remodeling in a ubiquitous planktonic photosymbiosis. *Current Biology* **29**, 968-978.e4 (2019).
32. Kumar, M. *et al.* Mixotrophic growth of a ubiquitous marine diatom. *Sci Adv* **10**, eado2623 (2024).
33. Zuñiga, C. *et al.* Genome-scale metabolic model for the green alga *Chlorella vulgaris* UTEX 395 accurately predicts phenotypes under autotrophic, heterotrophic, and mixotrophic growth conditions. *Plant Physiol* **172**, 589–602 (2016).
34. Allen, A. E. *et al.* Evolution and metabolic significance of the urea cycle in photosynthetic diatoms. *Nature* **473**, 203–207 (2011).
35. Bertrand, E. M. *et al.* Phytoplankton-bacterial interactions mediate micronutrient colimitation at the coastal Antarctic sea ice edge. *Proc Natl Acad Sci U S A* **112**, 9938–9943 (2015).
36. Rao, D. *et al.* Flexible B_{12} ecophysiology of *Phaeocystis antarctica* due to a fusion B_{12}–independent methionine synthase with widespread homologues. *Proc Natl Acad Sci U S A* **121**, e2204075121 (2024).

37. Vernette, C. *et al.* The Ocean Gene Atlas v2.0: online exploration of the biogeography and phylogeny of plankton genes. *Nucleic Acids Res* **50**, W516–W526 (2022).
38. Wu, M. *et al.* Manganese and iron deficiency in Southern Ocean *Phaeocystis antarctica* populations revealed through taxon-specific protein indicators. *Nat Commun* **10**, 3582 (2019).
39. Lampe, R. H., Hernandez, G., Lin, Y. Y. & Marchetti, A. Representative diatom and coccolithophore species exhibit divergent responses throughout simulated upwelling cycles. *mSystems* **6**, e00188-21 (2021).
40. Alexander, H. *et al.* Functional group-specific traits drive phytoplankton dynamics in the oligotrophic ocean. *Proc Natl Acad Sci U S A* **112**, E5972–E5979 (2015).
41. Bender, S. J. *et al.* Colony formation in *Phaeocystis antarctica*: Connecting molecular mechanisms with iron biogeochemistry. *Biogeosciences* **15**, 4923–4942 (2018).
42. Gutowska, M. A. *et al.* Globally important haptophyte algae use exogenous pyrimidine compounds more efficiently than thiamin. *mBio* **8**, e01459-17 (2017).
43. Smith, S. R. *et al.* Evolution and regulation of nitrogen flux through compartmentalized metabolic networks in a marine diatom. *Nat Commun* **10**, (2019).
44. Raymond, J. A. & Kim, H. J. Possible role of horizontal gene transfer in the colonization of sea ice by algae. *PLoS One* **7**, e35968 (2012).
45. Dorrell, R. G. *et al.* Convergent evolution and horizontal gene transfer in Arctic Ocean microalgae. *Life Sci Alliance* **6**, e202201833 (2023).
46. Wu, Z. *et al.* Empirical bayes analysis of sequencing-based transcriptional profiling without replicates. *BMC Bioinformatics* **11**, 564 (2010).
47. Wurch, L. L. *et al.* Transcriptional shifts highlight the role of nutrients in harmful brown tide dynamics. *Front Microbiol* **10**, 136 (2019).
48. Gann, E. R., Hughes, B. J., Reynolds, T. B. & Wilhelm, S. W. Internal nitrogen pools shape the infection of *Aureococcus anophagefferens* CCMP1984 by a giant virus. *Front Microbiol* **15**, e0226758 (2020).
49. Sandaa, R. A. *et al.* Adaptive evolution of viruses infecting marine microalgae (haptophytes), from acute infections to stable coexistence. *Biol Rev* **97**, 179–194 (2022).
50. Becht, E. *et al.* Dimensionality reduction for visualizing single-cell data using UMAP. *Nat Biotechnol* **37**, 38–47 (2018).
51. Brisbin, M. M. & Mitarai, S. Differential gene expression supports a resource-intensive, defensive role for colony production in the bloom-forming haptophyte, *Phaeocystis globosa*. *J Eukaryot Microbiol* **66**, 788–801 (2019).
52. Zhang, S. F., Zhang, K., Cheng, H. M., Lin, L. & Wang, D. Z. Comparative transcriptomics reveals colony formation mechanism of a harmful algal bloom species *Phaeocystis globosa*. *Sci Total Environ* **719**, 137454 (2020).
53. Zhu, J. *et al.* Mechanisms of *Phaeocystis globosa* blooms in the Beibu Gulf revealed by metatranscriptome analysis. *Harmful Algae* **124**, 102407 (2023).
54. Zhang, S. F. *et al.* Quantitative proteomic analysis reveals the key molecular events driving *Phaeocystis globosa* bloom and dissipation. *Int J Mol Sci* **23**, (2022).
55. Weng, L. C. *et al.* Nitrogen deprivation induces lipid droplet accumulation and alters fatty acid metabolism in symbiotic dinoflagellates isolated from *Aiptasia pulchella*. *Sci Rep* **4**, 4577 (2014).

56. Chokshi, K., Pancha, I., Ghosh, A. & Mishra, S. Nitrogen starvation-induced cellular crosstalk of ROS-scavenging antioxidants and phytohormone enhanced the biofuel potential of green microalga *Acutodesmus dimorphus*. *Biotechnol Biofuels* **10**, 60 (2017).
57. Glibert, P. M. *et al.* Pluses and minuses of ammonium and nitrate uptake and assimilation by phytoplankton and implications for productivity and community composition, with emphasis on nitrogen-enriched conditions. *Limnol Oceanogr* **61**, 165–197 (2016).
58. Smith, W. O., Carlson, C. A., Ducklow, H. W. & Hansell, D. A. Growth dynamics of *Phaeocystis antarctica*-dominated plankton assemblages from the Ross Sea. *Mar Ecol Prog Ser* **168**, 229–244 (1998).
59. Bradley, P. B. *et al.* Nitrogen uptake by phytoplankton and bacteria during an induced *Phaeocystis pouchetii* bloom, measured using size fractionation and flow cytometric sorting. *Aquat Microb Ecol* **61**, 89–104 (2010).
60. Wang, X., Wang, Y. & Smith, W. O. The role of nitrogen on the growth and colony development of *Phaeocystis globosa* (Prymnesiophyceae). *Eur J Phycol* **46**, 305–314 (2011).

SUPPLEMENTARY DATA LEGENDS:

Supplementary Data 1: Genome assembly characteristics of *Phaeocystis* spp. presented in this work. Reference-quality assemblies are shown with colored background, in colors corresponding to figures. Assembly statistics of a previously assembled *Emiliania* (*Gephyrocapsa*) huxleyi are shown for comparison. On the second sheet, basic completeness statistics are shown for MAGs from Delmont et al., 2022 (doi: 10.1016/j.xgen.2022.100123), and their inclusion in further analyses.

Supplementary Data 2: Repetitive element profile of *Phaeocystis* spp. reference genomes (nuclear assemblies). Data determined using REPET v.3, RepeatMasker, Tandem Repeats Finder, and DUST (below). Homology search based on protein structures detected a recombinase domain in ORFs in a few unclassified repeats and they were considered putative mobile elements referred to as "PutMobRec".

Supplementary Data 3: Datasets used in this study. Sampling cruises, their basic parameters, and references are listed. Only data for samples selected for this study are shown; we selected samples with good representation of relevant size fractions (e.g. Baltic Sea, Tara Oceans and Tara Arctic) and from the euphotic zone.

Supplementary Data 4: Functions associated with Self-Organizing Map super-clusters in NCOG data. Annotations with green background indicate clusters with relatively increased transcript proportion, whereas orange background indicates clusters with relatively decreased transcript proportion (Supplementary Fig. 10).

Supplementary Data 5: Up- and down-regulated functions according to ANCOM-BC associated with higher mitochondrial-to-plastid transcription. Data inferred for library size-normalized Phaglo1-mapping reads from stations with ln(MT/PT transcription) >-2 (high, n=30) and stations with ln(MT/PT transcription) between -4 and -2 (low, n=37) from stations between latitude 68°N and -56°S (polar stations excluded). Columns "mean" list the estimated log expression in high and low MT/PT transcription, q/lfc/se list the q (corrected) statistic, log-fold change, and standard error of relative expression, respectively. Pseudo-count_sensitivity marks if a KEGG orthology's differential expression passes the pseudo-count test of ANCOM-BC2; FALSE indicates a likely false positive. Columns H and I list descriptions and BRITE hierarchy for every significantly changed KEGG ortholog.

Supplementary Data 6: Phaeocystales adaptations in temperate and polar biotopes. Protein families (Pfams) and ORF clusters found differentially expressed or correlated with iron (Southern Ocean is iron-depleted) are listed. Sheet "top 1000" lists Pfams corresponding to top 1000 orthogroups with highest abundance (TPM) in each of the three biotopes (Arctic, Temperate, Southern Ocean) and their distribution. For instance, ATS marks the presence of a Pfam in the top

1000 in all three biotopes, AS marks a presence in polar biotopes (Arctic + Southern), and S marks an exclusive presence in the Southern Ocean biotope.

Supplementary Data 7: Transporters in *Phaeocystis* and other haptophytes. Table shows the A) counts and B) total gene model normalized counts of transporter genes assigned to families (i.e., per 100k genes). Phacord1 shows a higher normalized count of transporter genes than other *Phaeocystis*. Gene families and substrate classes explained on the third sheet.

Supplementary Data 8: Gene family evolution in Phaeocystales. Protein families found expanded (gain) or contracted (loss) by the evolution-informed maximum likelihood algorithm CAFE v4.0. Gains or losses per node were inferred based on terminal branch counts and their genetic distances from a multi-gene phylogeny constructed by PhyloFisher. KOGs were automatically or manually inferred from InterPro family annotation. RPM data represent mean reads per million across all biosamples for all genes with a given InterPro domain.

Supplementary Data 9: Putative homologies of ORFs predicted in endogenous NCLDV loci in *P. antarctica* (PaeNCLDV) and *P. globosa* (PgeNCLDV). Reference sequences only. Annotations determined using HH-suite and BLASTP, the total number of predicted ORFs are: PaeNCLDV type 1, 62; PaeNCLDV type 2, 56; PgeNCLDV, 46.

Supplementary Data 10: Statistically up- and down-regulated functions according to ASC posterior probability associated with various conditions. Columns B-D mark the direction of the change, log-e relative expression, and posterior probability, respectively. Columns E, G, H list BRITE hierarchy, symbol and description for every significantly changed KEGG ortholog (column F).

SUPPLEMENTARY FIGURES (starting next page)

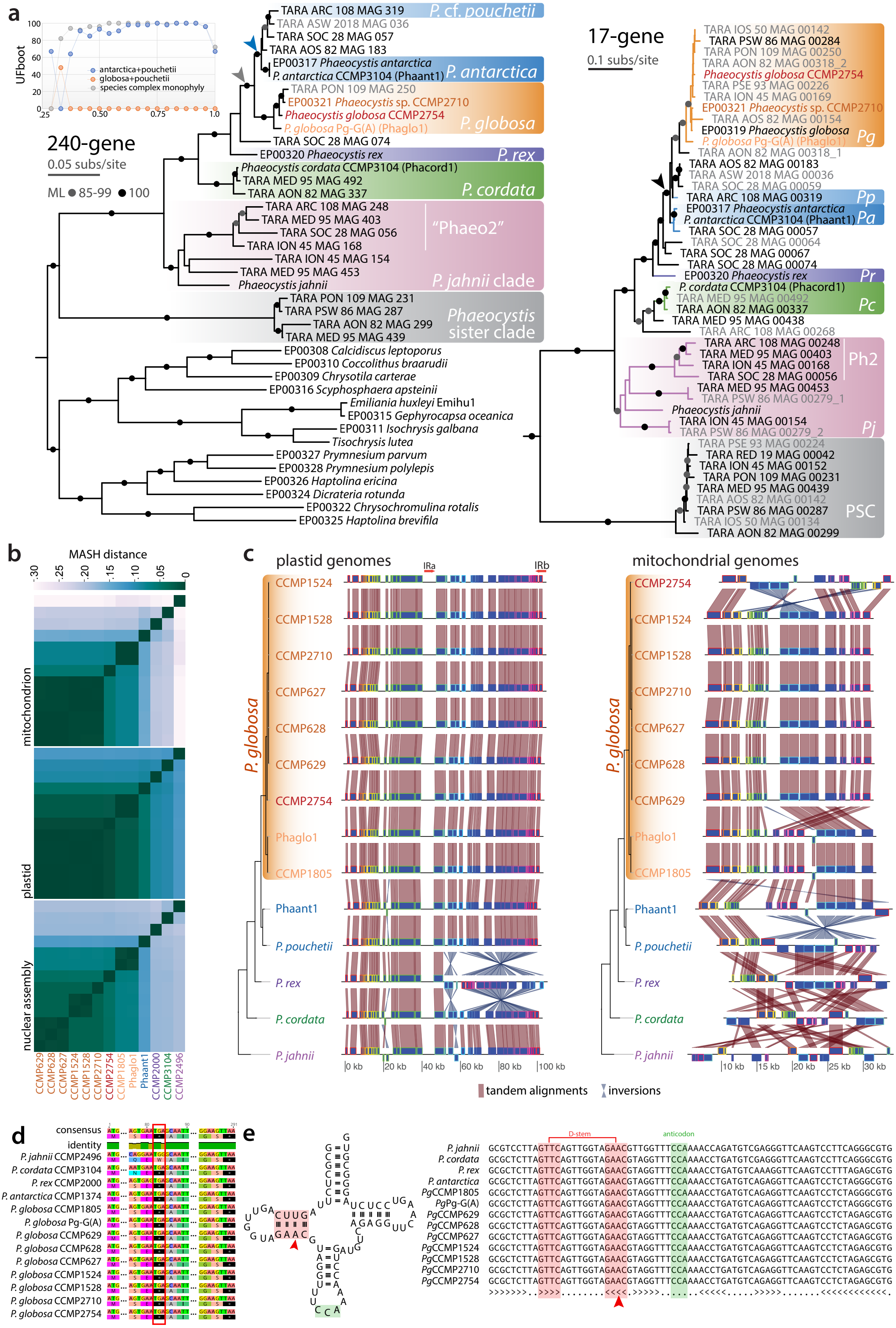
a
240-gene
0.05 subs/site
ML ● 85-99 ● 100
UFboot
antarctica+pouchetii
globosa+pouchetii
species complex monophyly
TARA ARC 108 MAG 319
TARA ASW 2018 MAG 036
TARA SOC 28 MAG 057
TARA AOS 82 MAG 183
P. cf. pouchetii
EP00317 Phaeocystis antarctica
P. antarctica CCMP3104 (Phaant1)
P. antarctica
TARA PON 109 MAG 250
EP00321 Phaeocystis sp. CCMP2710
Phaeocystis globosa CCMP2754
P. globosa Pg-G(A) (Phaglo1)
P. globosa
TARA SOC 28 MAG 074
EP00320 Phaeocystis rex
P. rex
Phaeocystis cordata CCMP3104 (Phacord1)
TARA MED 95 MAG 492
TARA AON 82 MAG 337
P. cordata
TARA ARC 108 MAG 248
TARA MED 95 MAG 403
TARA SOC 28 MAG 056
TARA ION 45 MAG 168
"Phaeo2"
TARA ION 45 MAG 154
TARA MED 95 MAG 453
Phaeocystis jahnii
P. jahnii clade
TARA PON 109 MAG 231
TARA PSW 86 MAG 287
TARA AON 82 MAG 299
TARA MED 95 MAG 439
Phaeocystis sister clade
EP00308 Calcidiscus leptoporus
EP00310 Coccolithus braarudii
EP00309 Chrysotila carterae
EP00316 Scyphosphaera apsteinii
Emiliania huxleyi Emihu1
EP00315 Gephyrocapsa oceanica
EP00311 Isochrysis galbana
Tisochrysis lutea
EP00327 Prymnesium parvum
EP00328 Prymnesium polylepis
EP00326 Haptolina ericina
EP00324 Dicrateria rotunda
EP00322 Chrysochromulina rotalis
EP00325 Haptolina brevifila
17-gene
0.1 subs/site
TARA IOS 50 MAG 00142
TARA PSW 86 MAG 00284
TARA PON 109 MAG 00250
TARA AON 82 MAG 00318_2
Phaeocystis globosa CCMP2754
TARA PSE 93 MAG 00226
TARA ION 45 MAG 00169
EP00321 Phaeocystis sp. CCMP2710
TARA AOS 82 MAG 00154
EP00319 Phaeocystis globosa
P. globosa Pg-G(A) (Phaglo1)
Pg
TARA AON 82 MAG 00318_1
TARA AOS 82 MAG 00183
TARA ASW 2018 MAG 00036
TARA SOC 28 MAG 00059
TARA ARC 108 MAG 00319
Pp
EP00317 Phaeocystis antarctica
P. antarctica CCMP3104 (Phaant1)
Pa
TARA SOC 28 MAG 00057
TARA SOC 28 MAG 00064
TARA SOC 28 MAG 00067
TARA SOC 28 MAG 00074
EP00320 Phaeocystis rex
Pr
P. cordata CCMP3104 (Phacord1)
TARA MED 95 MAG 00492
TARA AON 82 MAG 00337
Pc
TARA MED 95 MAG 00438
TARA ARC 108 MAG 00268
TARA ARC 108 MAG 00248
TARA MED 95 MAG 00403
TARA ION 45 MAG 00168
TARA SOC 28 MAG 00056
Ph2
TARA MED 95 MAG 00453
TARA PSW 86 MAG 00279_1
Phaeocystis jahnii
TARA ION 45 MAG 00154
TARA PSW 86 MAG 00279_2
Pj
TARA PSE 93 MAG 00224
TARA RED 19 MAG 00042
TARA ION 45 MAG 00152
TARA PON 109 MAG 00231
TARA MED 95 MAG 00439
TARA AOS 82 MAG 00142
TARA PSW 86 MAG 00287
TARA IOS 50 MAG 00134
TARA AON 82 MAG 00299
PSC
b
MASH distance
mitochondrion
plastid
nuclear assembly
CCMP629
CCMP628
CCMP627
CCMP1524
CCMP1528
CCMP2710
CCMP2754
CCMP1805
Phaglo1
Phaant1
CCMP2000
CCMP3104
CCMP2496
c
plastid genomes
IRa
IRb
P. globosa
CCMP1524
CCMP1528
CCMP2710
CCMP627
CCMP628
CCMP629
CCMP2754
Phaglo1
CCMP1805
Phaant1
P. pouchetii
P. rex
P. cordata
P. jahnii
0 kb
20 kb
40 kb
60 kb
80 kb
100 kb
mitochondrial genomes
CCMP2754
CCMP1524
CCMP1528
CCMP2710
CCMP627
CCMP628
CCMP629
Phaglo1
CCMP1805
Phaant1
P. pouchetii
P. rex
P. cordata
P. jahnii
10 kb
15 kb
20 kb
25 kb
30 kb
tandem alignments
inversions
d
consensus
identity
P. jahnii CCMP2496
P. cordata CCMP3104
P. rex CCMP2000
P. antarctica CCMP1374
P. globosa CCMP1805
P. globosa Pg-G(A)
P. globosa CCMP629
P. globosa CCMP628
P. globosa CCMP627
P. globosa CCMP1524
P. globosa CCMP1528
P. globosa CCMP2710
P. globosa CCMP2754
e
D-stem
anticodon
P. jahnii
P. cordata
P. rex
P. antarctica
PgCCMP1805
PgPg-G(A)
PgCCMP629
PgCCMP628
PgCCMP627
PgCCMP1524
PgCCMP1528
PgCCMP2710
PgCCMP2754

Supplementary Fig. 1 Phylogenetic relationship of *Phaeocystis* draft genomes and an alternative genetic code in *Phaeocystis* spp. plastids. **a** Multi-gene maximum likelihood phylogenies based on 240 nuclear genes (65,716 amino acid positions; ML represents both ultra-fast bootstrap and SH-aLRT being in the given range) and 17 nuclear genes (14,953 amino acid positions), stable over a large range of fastest site removal replicates (upper left, showing ultra-fast bootstrap support for the branchings in question; arrowheads in the tree mark the branches tested). This strongly supports the monophyly of Arctic and Antarctic species. The data also support the separation of three *P. globosa* genotype, exemplified by CCMP2710, CCMP2754, and Pg-G(A) (Phaglo1), respectively. MAGs in grey were omitted from later biogeographic analyses due to their lower completeness or redundancy. The time-tree corresponding to the 17-gene phylogeny is available at iTOL (https://itol.embl.de/tree/9712447341355417417 17954). **b** Mash genetic distance, an approximation of average nucleotide identity, is shown for the three genetic compartments. Note the higher genetic distances of the nuclear assemblies of the *P. globosa* genotype 1 accessions compared to the respective mitochondrial genome distances, suggestive of ongoing ecological/reproductive differentiation between genotypes 1-3. **c** Organellar genome colinearity in *Phaeocystis* spp. and genome-wide genetic distance of *Phaeocystis* draft genomes. Whole genomes were aligned by Mauve, visualized by genoPlotR. Red arrows denote inverted repeats of plastid genomes. Note that in the mitochondrial panel *P. globosa* genotype 2 (CCMP2754) is on top to highlight the rearrangements between genotypes 1-3. This suggests a population or sub-species structure in *P. globosa*. **d** Despite being initially labeled as a pseudogene due to an in-frame UGA, the plastid gene *ycf20* exhibits strong conservation across these genomes and likely utilizes tRNA-Trp to compensate. The nucleotide alignment of *ycf20* shows that UGA likely encodes for Trp in *Phaeocystis* spp., highlighted by the red rectangle. UGA stop codons are absent in other genes; *P. jahnii* has 2 UGA, 98 UAA, and 13 UAG stop codons; other *Phaeocystis* have no UGA as stops, and on average 82 UAA and 27 UAG stop codons. Stop-to-Trp codon reassignment is also corroborated by a Hirsh mutation in the D-stem of tRNA-W(CCA), which is conserved in *Phaeocystis* spp. (arrowhead in **e**).

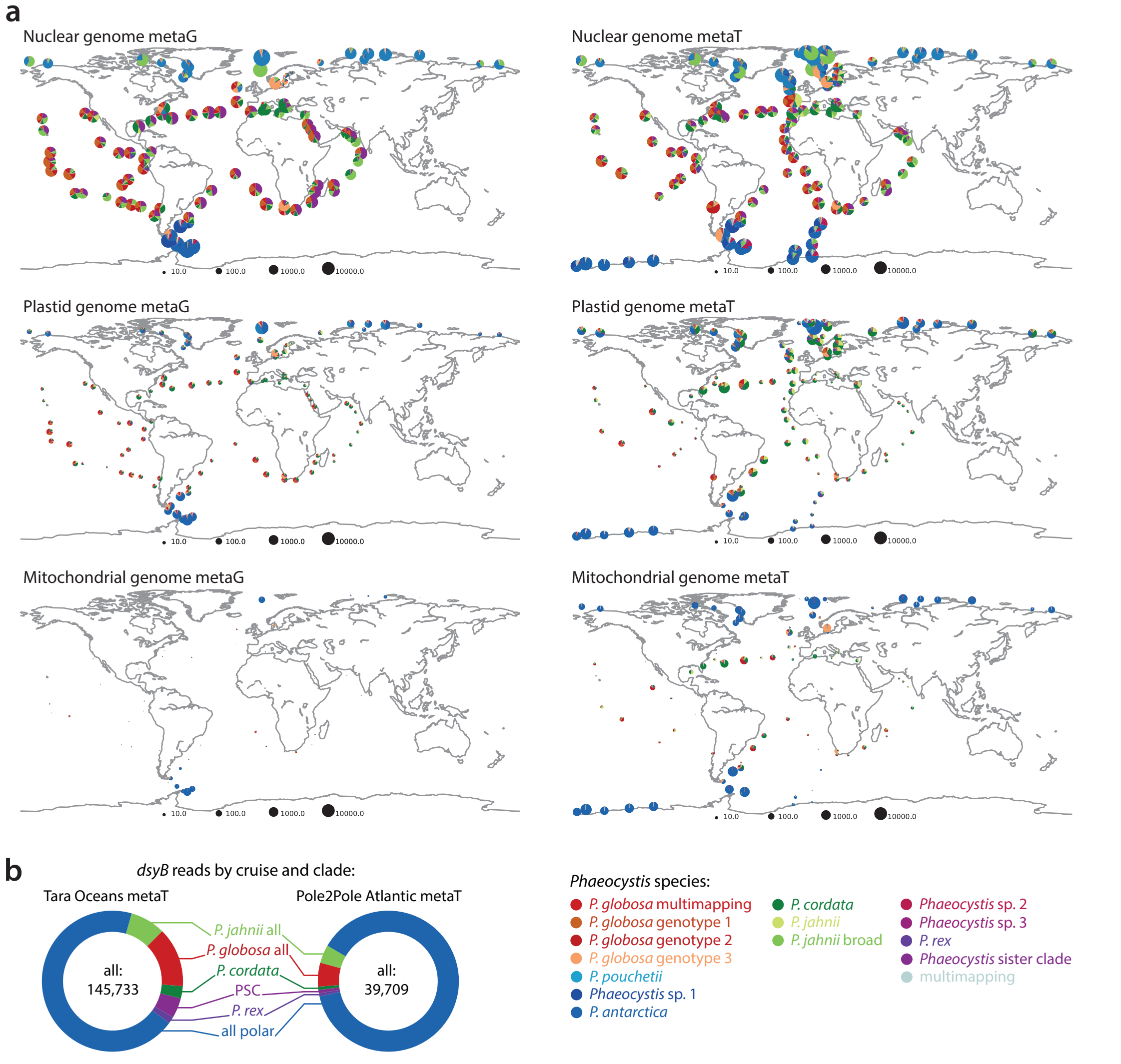

Supplementary Fig. 2 Comparison of read-mapping to genetic compartments of *Phaeocystis* spp. across worldwide stations. **a** Each taxon's fraction of the total number of mapped reads per station is colored according to the color legend. Data for *P. globosa* accessions were pooled according to their phylogenetic relationship (Extended Data Fig. 1). Note the different occurrence of not only *Phaeocystis* spp. but also *P. globosa* genotypes apparent across stations. Station totals are normalized to millions of reads sequenced (RPM) and shown in log scale, with the size legend below each panel. Only a selection of Pole2Pole Atlantic transect stations is shown for clarity. Plastid genomes recruited substantially more metaT reads than metaG reads, consistent with high photosynthetic plastid transcription. **b** Contribution of each clade to all reads mapping to all *dsyB* genes identified in the genome assemblies and MAGs (normalized to query Phaant1_3838 coverage). Centers show the total number of reads mapping to found Phaeocystales *dsyB* across all sampled stations.

a

	P. globosa genotype 1	*P. globosa* genotype 2	*P. globosa* genotype 3	*P. pouchetii*	*P. antarctica*	*P. rex*	*P. cordata*	*P. jahnii* broad	*Phaeocystis* sister clade
	S	S	S	S	S	S	S	S	S
s(log(Chl_a))	***	***	***	***	***			**	***
s(Iron_5m)	***	***	**	***	***	**			***
s(Nitrate_5m)				***	***		***		***
s(log(Ammonium_5m))	***	***	***	***	***	*			***
s(diatom_abund)	*	*	***	***	***	**	***		***
s(dinoflagellate_abund)									
s(Temperature)	***	***	***	***	***	**	***		***
s(Distance_coast)	***	***	*			***			***
Deviance explained [%]	74.0	75.7	61.6	92.2	85.6	48.9	49.1	37.1	71.2

Significance codes: *** 0-0.001 ** 0.001-0.01 * 0.01-0.05

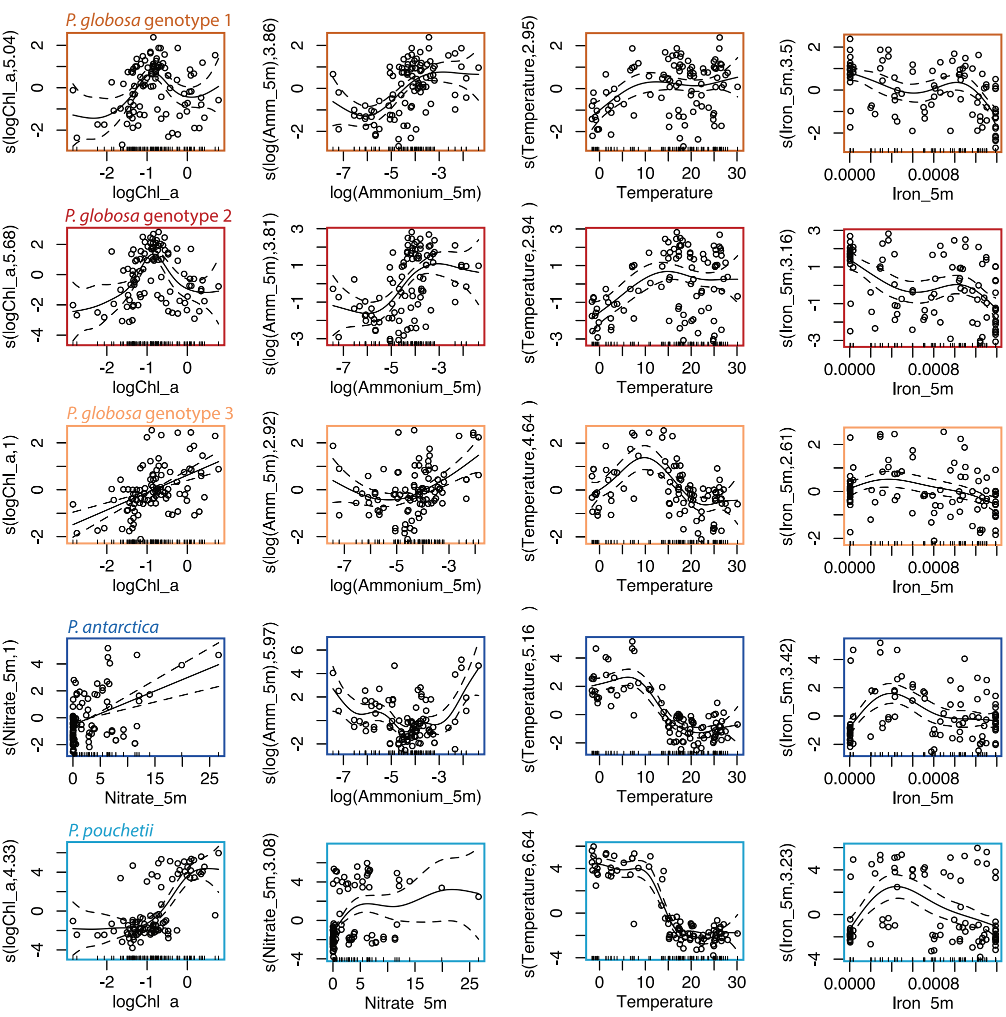

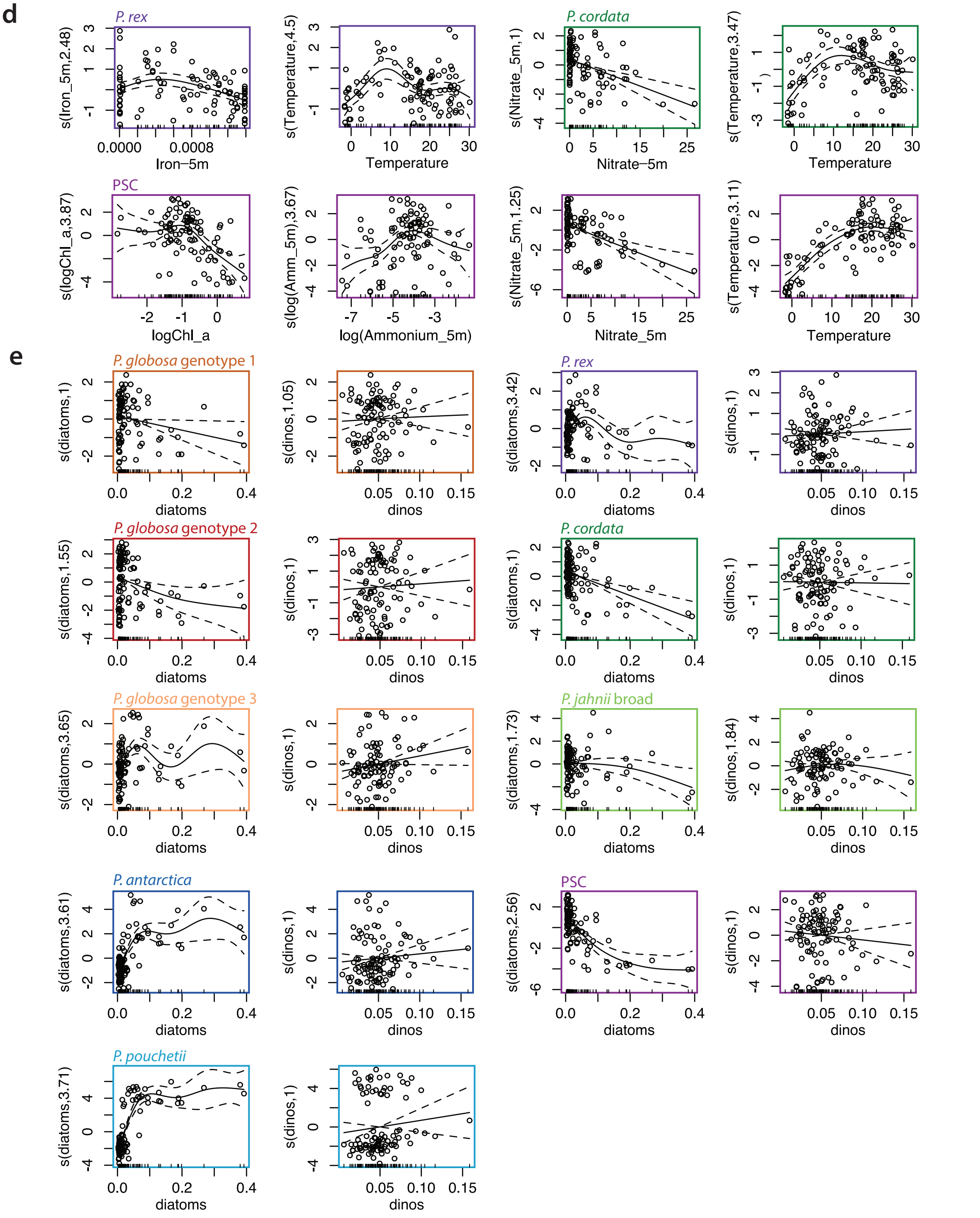

Supplementary Fig. 3 Generalized additive models analysis of *Phaeocystis* spp. metaT abundance in small size fractions (<20μ m) versus environmental parameters. **a** Result summary of the approximate significance of smooth terms, with significant values shown as codes. The smoothing parameter was determined by the restricted ML method (method="REML") with a maximum of 5 basis functions (k=5). Lineages with low global abundance were omitted from the analysis, and so were data for large size fractions due to low numbers of samples with sufficient abundance data. The deviance explained pertains to runs with all independentparameters included. Example environmental parameter fits: **b** *P. globosa* genotypes; **c** polar *Phaeocystis*; **d** non-colony forming *Phaeocystis*. **e** Fits with metaT abundace of diatoms and dinoflagellates (normalized MATOU clade abundance per station and depth).

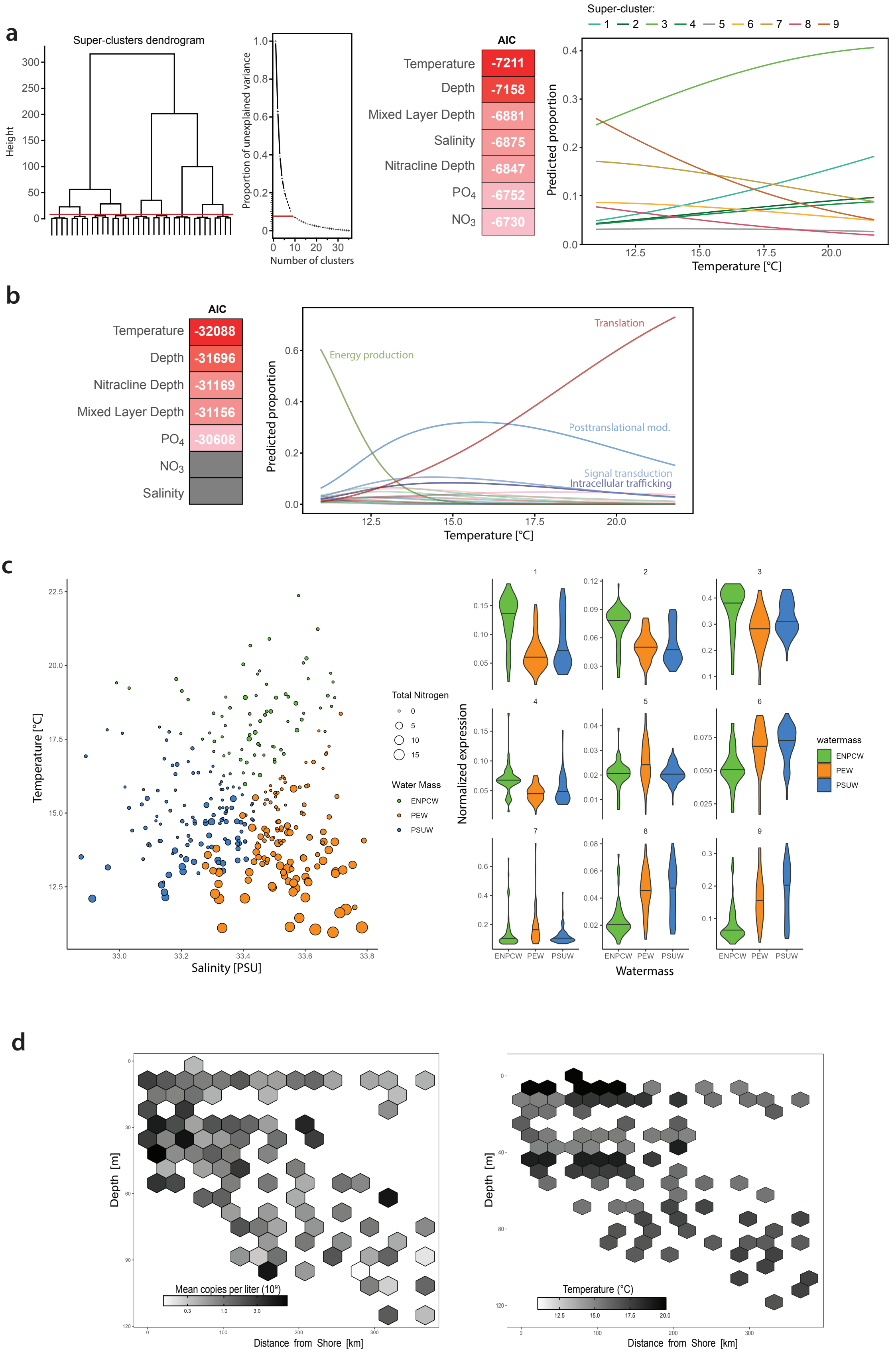

a
Super-clusters dendrogram
Height
Proportion of unexplained variance
Number of clusters
AIC
Temperature -7211
Depth -7158
Mixed Layer Depth -6881
Salinity -6875
Nitracline Depth -6847
PO4 -6752
NO3 -6730
Super-cluster: 1 2 3 4 5 6 7 8 9
Predicted proportion
Temperature [°C]
b
AIC
Temperature -32088
Depth -31696
Nitracline Depth -31169
Mixed Layer Depth -31156
PO4 -30608
NO3
Salinity
Translation
Energy production
Posttranslational mod.
Signal transduction
Intracellular trafficking
Predicted proportion
Temperature [°C]
c
Temperature [°C]
Salinity [PSU]
Total Nitrogen
Water Mass
ENPCW
PEW
PSUW
Normalized expression
watermass
Watermass
d
Depth [m]
Distance from Shore [km]
Mean copies per liter (10^9)
Temperature (°C)

Supplementary Fig. 4 Metatranscriptomic changes of *Phaeocystis* spp. in California Current Ecosystem data (NOAA CalCOFI Ocean Genomics Project) . An available metaT assembly was used for statistical analyses to account for sequences not covered by our strain and MAG genomic data. Phaeocystales open reading frames were identified using Lineage Probability Index (LPI) and clustered to orthologous groups (see Methods). **a** Self-organizing maps (SOM) were applied to metaT abundances of these orthogroups, and the dendrogram represents hierarchical clustering of these maps. The corresponding elbow plot describes the proportion of residual variance not explained by clustering to super-clusters. Akaike Information Criterion (AIC) was used to infer likelihood of environmental parameters driving any variable transcription, followed by Dirichlet regression to estimate cluster relative abundance across a range of parameters. The heatmap shows environmental variables ranked by their AIC likelihood, with the Dirichlet regression on the right shown across a range of temperatures using orthogroups having a Pfam annotation. Pfams associated with the clusters with relatively increased transcript proportion (1-4) largely related to nuclear and vesicular transport, replication, expression and protein homeostasis, photosynthesis-related metabolism, amino acid synthesis, and fatty acid synthesis and desaturation, suggestive of anabolic changes (Supplementary Table S4). In contrast, clusters with relatively decreased transcript proportion (7-8) contained few exclusive biological functions, most remarkably cyclins, cyclin-dependent kinases, and MPV17, a mitochondrial DNA copy number and maintenance protein, suggesting a switch from mitochondrial to plastid-driven metabolism over this temperature (and depth) gradient. **b** As in **a** but categories represented by KOG functional annotation. Note that photosynthesis proteins are not captured well by the KOG annotation tool, therefore "Energy production" is mostly represented by mitochondrial respiratory chain orthogroups. For clarity, minor functional categories are shown in lower opacity. **c** Temperature, salinity and total nitrogen of the samples collected across three main water masses in the California Current Ecosystem are shown on the left, whereas the violin plots (with median lines) on the right show the transcriptomic abundance profiles of the 9 super-clusters from A in these three distinctive water masses (ENPCW, Eastern North Pacific Central Water; PEW, Pacific Equatorial Water; PSUW, Polar SUbarctic Water). **d** Binning density plot of mean *Phaeocystis* transcript per liter over distance from shore and depth, side by side temperature over distance and depth.

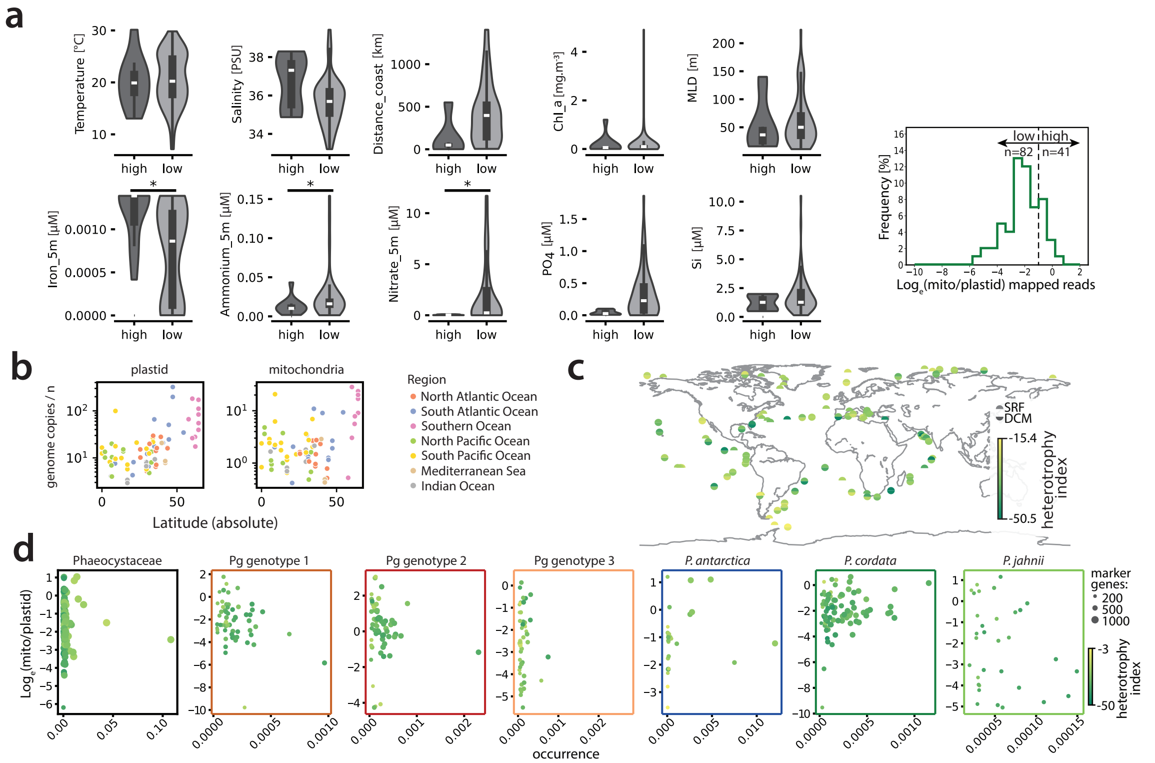

Supplementary Fig. 5 High mitochondrial transcription in some *Phaeocystis* spp. is linked to altered environmental conditions. **a** The distribution of environmental parameters for stations with high vs. low mitochondrial-to-plastid read coverage ratio in *P. cordata* . The lower threshold for "high coverage ratio" was ln=-3 for *P. cordata*, chosen to distinguish the two modes; compare with the histogram on the right (threshold marked with a dashed line). Statistically significant difference in low-iron/low-nitrogen areas is marked by asterisks (Benjamini/Hochberg-corrected two-sided Mann-Whitney U (n_1=41, n_2=82), Vargha-Delaney A; iron: U=2449, *p*=5.71e-05, A=0.61 [95% CI: 0.54, 0.74]; ammonium: U=1226, *p*=1.47e-02, A=0.42 [95% CI: 0.28, 0.50]; nitrate: U=872, *p*=4.3e-05, A=0.34 [95% CI: 0.25, 0.48]). The box and whisker plots show median, IQR and 1.5*IQR values, respectively. MLD, Mixed Layer Depth. **b** *Phaeocystis* organellar genome copies per haploid genome, normalized to nuclear single copy genes. **c** Heterotrophy index, calculated from detectable Phaeocystales MATOU unigenes occurrence in Tara Oceans stations, varies from values consistent with photoautotrophic behavior (more negative, green) to values consistent with mixotrophy (closer to zero, yellow). **d** Heterotrophy index as a function of occurrence and mito/plastid expression ratio for Phaeocystales, high-mitochondrial expression lineages (*P. globosa* genotype 2, *P. cordata* , and *P. antarctica*), and low-mitochondrial expression

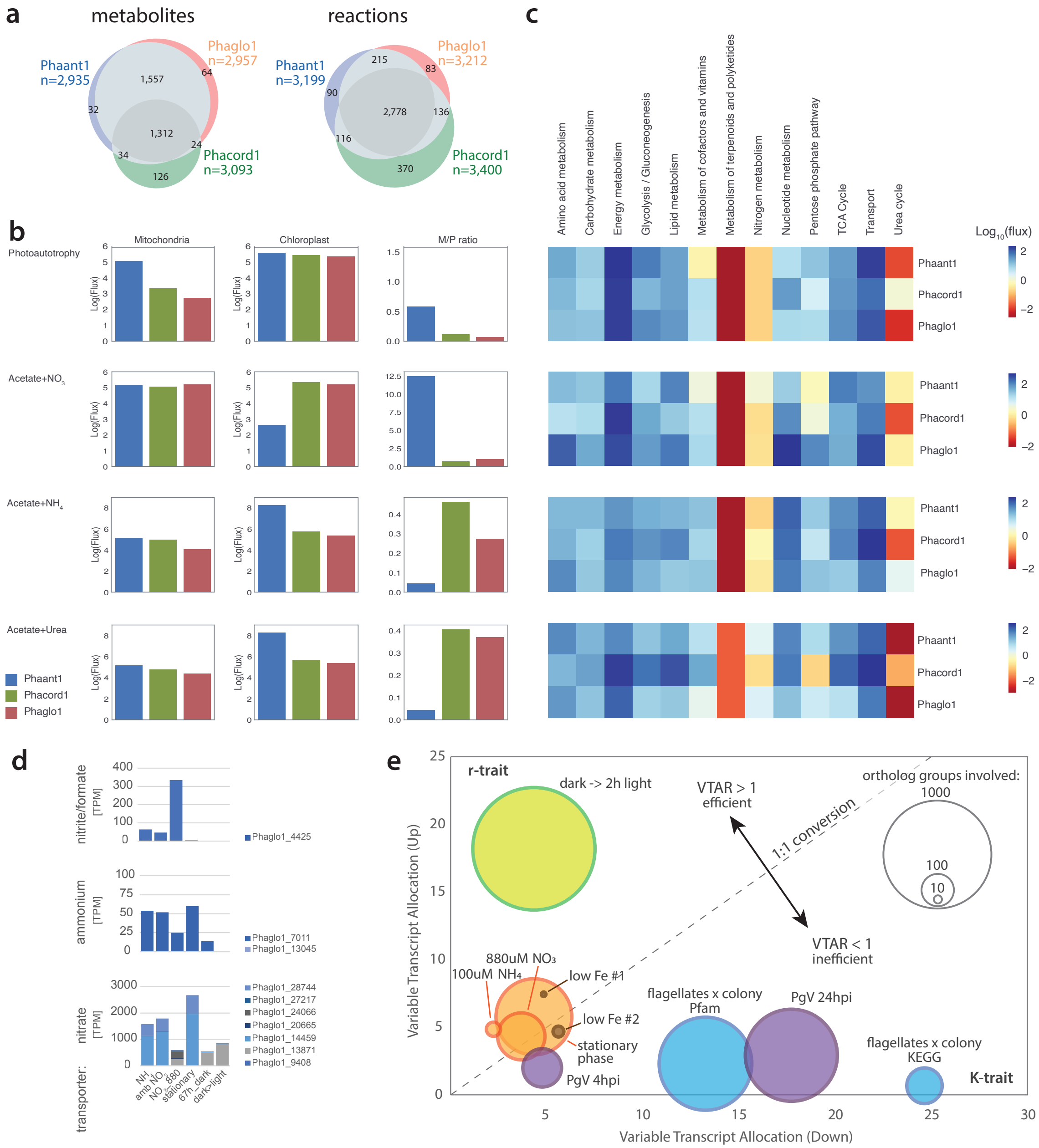

Supplementary Fig. 6 Comparative analysis of metabolic flux distributions in *P. antarctica*, *P. globosa*, and *P. cordata*. Transcriptomic changes triggered by various experimental conditions with *P. globosa* Pg-G(A). **a** Shared and unique reactions and metabolites between three genome-scale metabolic models (GEMs). **b** Metabolic flux (mmol.gDW^{-1}.h^{-1}) distribution among plastids and mitochondria under phototrophic and mixotrophic conditions (acetate+nitrogen source), and their mitochondrial-to-plastid ratio (M/P). Note different ranges on y-axes. **c** Metabolic fluxes through various pathways within the network corresponding to trophic modes in B (to their left). Higher flux values indicate increased activity within a particular pathway, while lower values signify reduced activity. Note the flux data is $\log_{10}$ transformed, see scale bars on the right. **d** Expression responses of identified nitrogen compound transporters to nitrogen-related treatments. NH_4 - ammonium supplementation; amb NO_3 - ambient nitrate level (0.37 µM nitrate, negative control); NO_3_880 - nitrate supplementation at 880 µM; stationary - nitrate supplemented culture entering stationary phase 18 days post inoculation; 67h_dark - dark growth for 67 hours; dark>light - dark-grown cells exposed to light for 2 hours. Where there are multiple transporters of the same family, their gene identifiers are shown in shades of color. TPM, transcripts per million. **e** Variable Transcript Allocation Ratio (VTAR) for the transcriptomic comparisons, colored by treatment. VTAR characterizes an organism's ability to transcriptionally respond to environmental cues, i.e. to what extent new transcription contributes to this response. The plot shows that dark-to-light transition effectively triggers new transcription, whereas nitrogen and iron treatments have moderate effect, and colony formation and PgV infection 24 hours post infection result in significantly decreased transcription only. Only orthologous groups found significantly changed by ASC

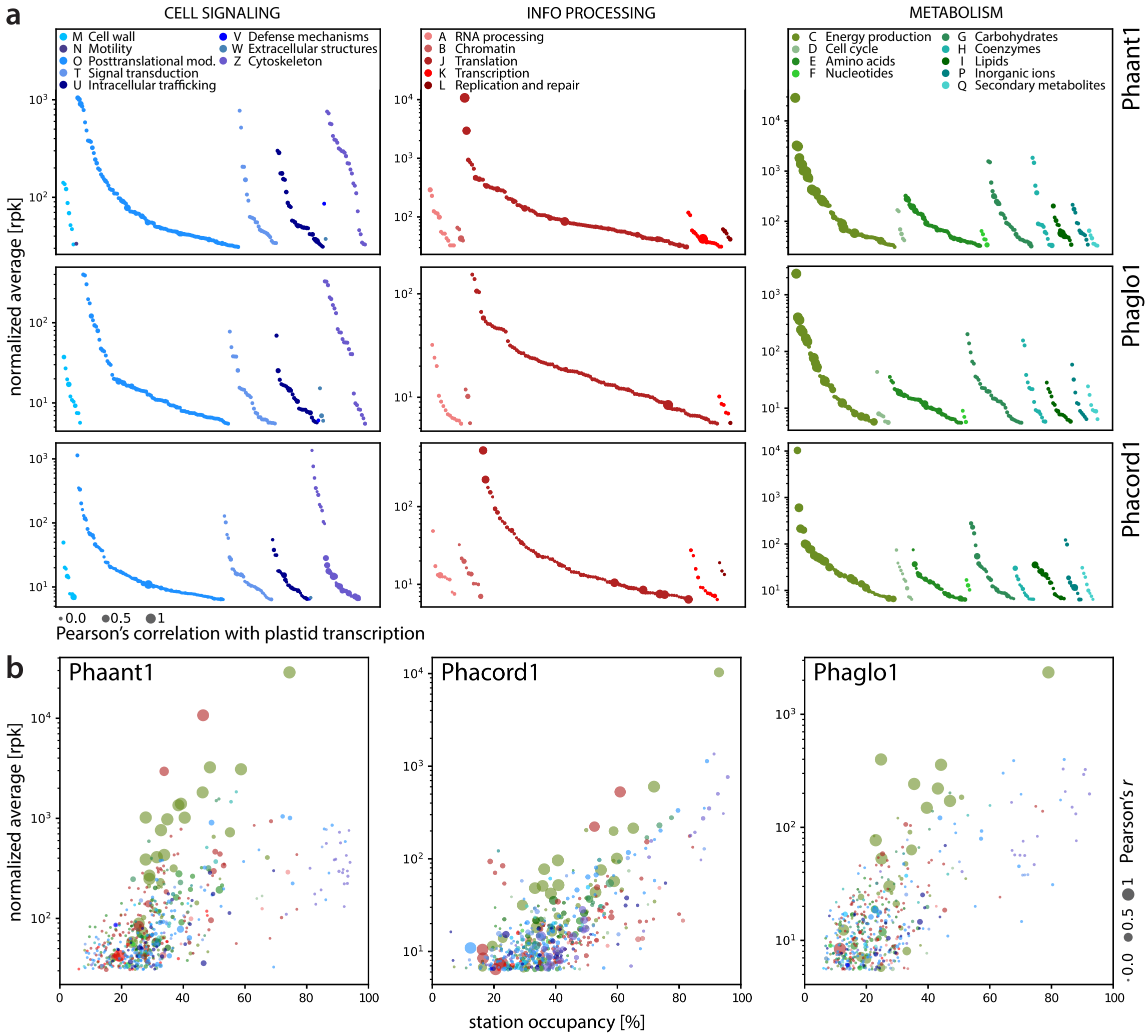

Supplementary Fig. 7 Most abundantly transcribed genes of *P. antarctica* Phaant1, *P. globosa* Phaglo1, and *P. cordata* Phacord1. **a** Data points, representing average normalized abundance of each gene, are colored by KEGG Orthology Group (KOG) term, and sized by the degree of correlation with plastid-mapping reads across all Tara station data. Functions of 1,000 most highly abundant genes are shown for each genome. rpk, reads per kilobase (of transcript length). **b** Scatter plots of mean normalized expression vs. station occupancy. Marker sizes represent correlation with plastid transcription. rpk, reads per kilobase.

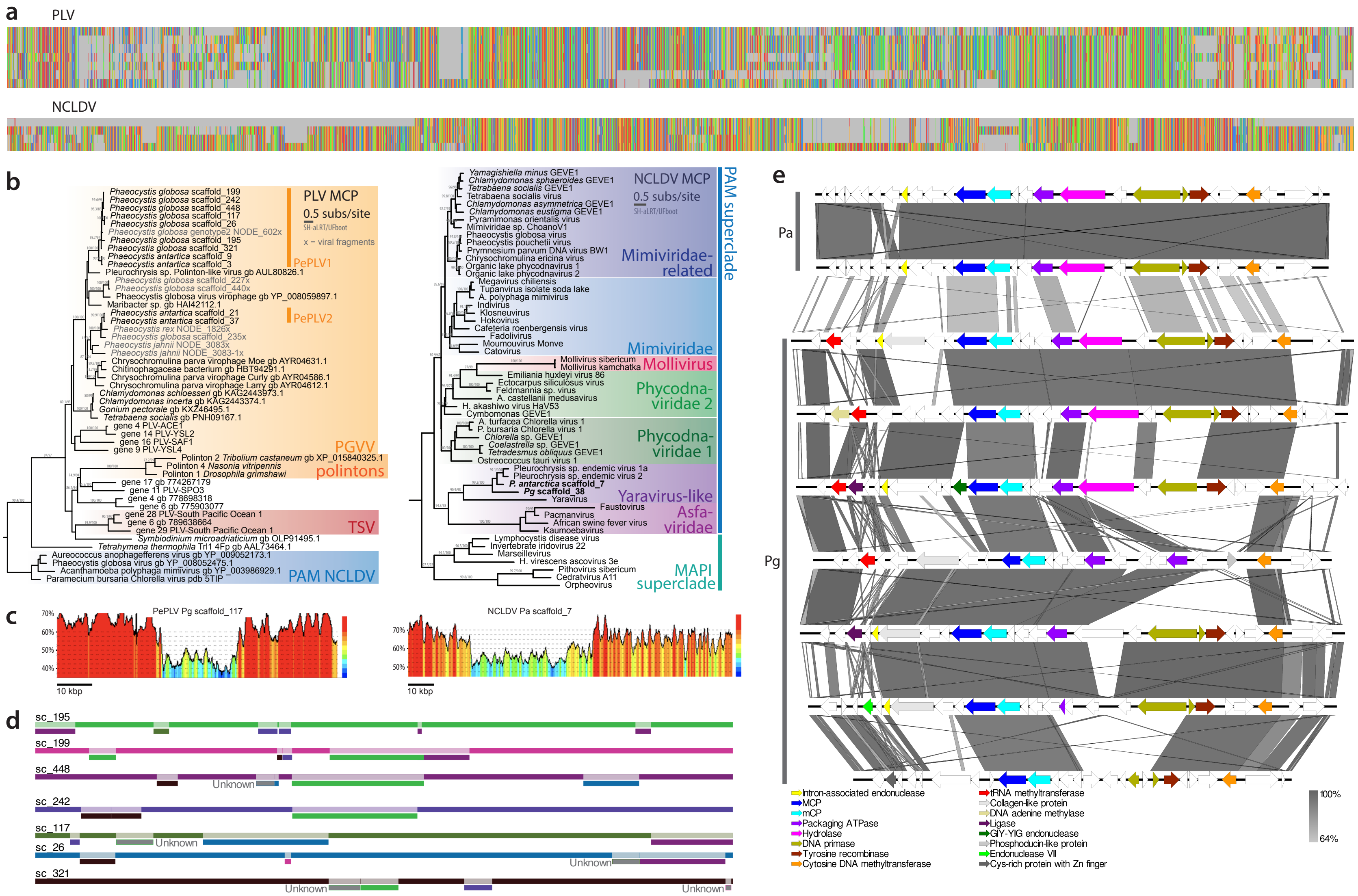

a
PLV
NCLDV
b
PLV MCP
0.5 subs/site
x – viral fragments
PePLV1
PePLV2
PGVV
polintons
TSV
PAM NCLDV
NCLDV MCP
0.5 subs/site
PAM superclade
Mimiviridae-related
Mimiviridae
Mollivirus
Phycodnaviridae 2
Phycodnaviridae 1
Yaravirus-like
Asfaviridae
MAPI superclade
c
PePLV Pg scaffold_117
NCLDV Pa scaffold_7
10 kbp
d
sc_195
sc_199
sc_448
sc_242
sc_117
sc_26
sc_321
Unknown
e
Pa
Pg
Intron-associated endonuclease
MCP
mCP
Packaging ATPase
Hydrolase
DNA primase
Tyrosine recombinase
Cytosine DNA methyltransferase
tRNA methyltransferase
Collagen-like protein
DNA adenine methylase
Ligase
GIY-YIG endonuclease
Phosphoducin-like protein
Endonuclease VII
Cys-rich protein with Zn finger
100%
64%

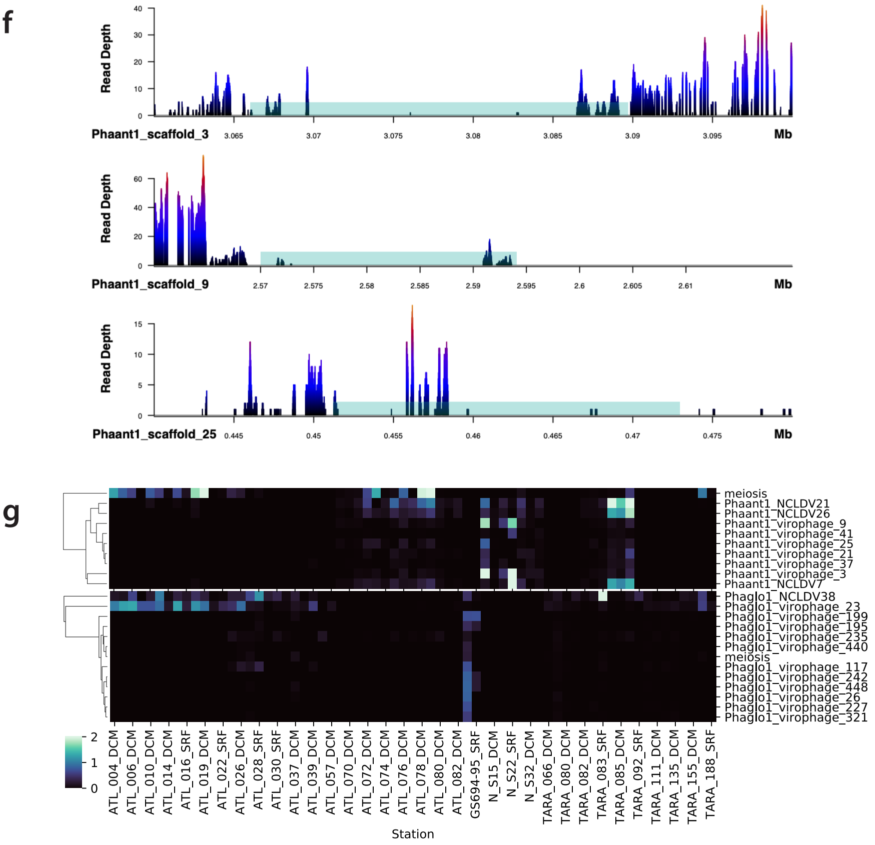

Supplementary Fig. 8 Phaeocystis nucleocytoplasmic large DNA virus (NCLDV) and polinton-like virophage (PLV) sequences integrated into the genomes of *Phaeocystis* spp. **a** Full-length alignments of endogenous viral sequences in *P. antarctica* (Pa) and *P. globosa* (Pg). **b** Phylogenies of Phaeocystis endogenous PLV (PePLV) and NCLDV based on major capsid protein (MCP). Only loci of putatively full-length viruses are shown, with the exception of PLV MCP sequences from viral fragments found in other *Phaeocystis* species (marked with x, in grey), suggesting PLV infections but not full-length insertions are common for most Phaeocystales. Branch supports are shown where SH-aLRT or UFboot >90. **c** PePLV1 and NCLDV have relatively low GC content (ranging 40-48%) compared to Phaeocystis genomes and appear as large GC-poor islands. Examples of GC-poor islands in Pg scaffold_117 and Pa scaffold_7. **d** Predicted recombination events among Pg PePLV1 copies. **e** Annotation and collinearity of PePLV1 Pa and Pg copies. **f** Example read depth from SAMEA2621523 (TARA_085, surface layer fraction 0.8-5 µm). Virophage similarity regions are highlighted by shaded bars. **g** Abundance heatmap (normalized reads per million reads) of identified Phaant1 and Phaglo1 viral sequences from metaT data showing a coincidence of reads mapping to PLV, NCLDV, and meiotic genes (MER3, MND1, HOP1, DMC1) loci at some stations, suggesting potential biological interactions between the three organisms.
