# Supplementary material for: Genome-resolved biogeography of Phaeocystales, cosmopolitan bloom-forming algae
Source: Nat Commun. 2025 Sep 29;16:8559. doi: 10.1038/s41467-025-63565-1 (PMC12480563; doi:10.1038/s41467-025-63565-1)
Supplement: Supplementary file 2 — Description of Additional Supplementary Files [file 41467_2025_63565_MOESM2_ESM.pdf]

Description of Additional Supplementary Files

Supplementary Data 1: Genome assembly characteristics of *Phaeocystis* spp. presented in this work. Reference-quality assemblies are shown with colored background, in colors corresponding to figures. Assembly statistics of a previously assembled *Emiliania* (*Gephyrocapsa*) *huxleyi* are shown for comparison. On the second sheet, basic completeness statistics are shown for MAGs from Delmont et al., 2022 (doi: 10.1016/j.xgen.2022.100123), and their inclusion in further analyses.

Supplementary Data 2: Repetitive element profile of *Phaeocystis* spp. reference genomes (nuclear assemblies). Data determined using REPET v.3, RepeatMasker, Tandem Repeats Finder, and DUST (below). Homology search based on protein structures detected a recombinase domain in ORFs in a few unclassified repeats and they were considered putative mobile elements referred to as “PutMobRec”.

Supplementary Data 3: Datasets used in this study. Sampling cruises, their basic parameters, and references are listed. Only data for samples selected for this study are shown; we selected samples with good representation of relevant size fractions (e.g. Baltic Sea, Tara Oceans and Tara Arctic) and from the euphotic zone.

Supplementary Data 4: Functions associated with Self-Organizing Map super-clusters in NCOG data. Annotations with green background indicate clusters with relatively increased transcript proportion, whereas orange background indicates clusters with relatively decreased transcript proportion (Supplementary Fig. 10).

Supplementary Data 5: Up- and down-regulated functions according to ANCOM-BC associated with higher mitochondrial-to-plastid transcription. Data inferred for library size-normalized Phaglo1-mapping reads from stations with ln(MT/PT transcription) >-2 (high, n=30) and stations with ln(MT/PT transcription) between -4 and -2 (low, n=37) from stations between latitude 68°N and -56°S (polar stations excluded). Columns "mean" list the estimated log expression in high and low MT/PT transcription, q/lfc/se list the q (corrected) statistic, log-fold change, and standard error of relative expression, respectively. Pseudo-count_sensitivity marks if a KEGG orthology's differential expression passes the pseudo-count test of ANCOM-BC2; FALSE indicates a likely false positive. Columns H and I list descriptions and BRITE hierarchy for every significantly changed KEGG ortholog.

Supplementary Data 6: Phaeocystales adaptations in temperate and polar biotopes. Protein families (Pfams) and ORF clusters found differentially expressed or correlated with iron (Southern Ocean is iron-depleted) are listed. Sheet "top 1000" lists Pfams corresponding to top 1000 orthogroups with highest abundance (TPM) in each of the three biotopes (Arctic, Temperate, Southern Ocean) and their distribution. For instance, ATS marks the presence of a Pfam in the top 27 1000 in all three biotopes, AS marks a presence in polar biotopes (Arctic + Southern), and S marks an exclusive presence in the Southern Ocean biotope.

Supplementary Data 7: Transporters in *Phaeocystis* and other haptophytes. Table shows the A) counts and B) total gene model normalized counts of transporter genes assigned to families (i.e., per 100k genes). Phacord1 shows a higher normalized count of transporter genes than other *Phaeocystis*. Gene families and substrate classes explained on the third sheet.

Supplementary Data 8: Gene family evolution in Phaeocystales. Protein families found expanded (gain) or contracted (loss) by the evolution-informed maximum likelihood algorithm CAFE v4.0. Gains or losses per node were inferred based on terminal branch counts and their genetic distances from a multi-gene phylogeny constructed by PhyloFisher. KOGs were automatically or manually inferred from InterPro family annotation. RPM data represent mean reads per million across all biosamples for all genes with a given InterPro domain.

Supplementary Data 9: Putative homologies of ORFs predicted in endogenous NCLDV loci in *P. antarctica* (PaeNCLDV) and *P. globosa* (PgeNCLDV). Reference sequences only. Annotations determined using HH-suite and BLASTP, the total number of predicted ORFs are: PaeNCLDV type 1, 62; PaeNCLDV type 2, 56; PgeNCLDV, 46.

Supplementary Data 10: Statistically up- and down-regulated functions according to ASC posterior probability associated with various conditions. Columns B-D mark the direction of the change, log-e relative expression, and posterior probability, respectively. Columns E, G, H list BRITE hierarchy, symbol and description for every significantly changed KEGG ortholog (column F).
