# Supplementary material for: Genome-resolved biogeography of Phaeocystales, cosmopolitan bloom-forming algae
Source: Nat Commun. 2025 Sep 29;16:8559. doi: 10.1038/s41467-025-63565-1 (PMC12480563; doi:10.1038/s41467-025-63565-1)
Supplement: Supplementary file 13 — Reporting Summary [file 41467_2025_63565_MOESM13_ESM.pdf]

nature portfolio

Corresponding author(s): Andrew E. Allen

Last updated by author(s): Jul 31, 2025

Reporting Summary

Nature Portfolio wishes to improve the reproducibility of the work that we publish. This form provides structure for consistency and transparency in reporting. For further information on Nature Portfolio policies, see our Editorial Policies and the Editorial Policy Checklist.

Statistics

For all statistical analyses, confirm that the following items are present in the figure legend, table legend, main text, or Methods section.

n/a	Confirmed	
☐	☒	The exact sample size (*n*) for each experimental group/condition, given as a discrete number and unit of measurement
☐	☒	A statement on whether measurements were taken from distinct samples or whether the same sample was measured repeatedly
☐	☒	The statistical test(s) used AND whether they are one- or two-sided<br>*Only common tests should be described solely by name; describe more complex techniques in the Methods section.*
☐	☒	A description of all covariates tested
☐	☒	A description of any assumptions or corrections, such as tests of normality and adjustment for multiple comparisons
☐	☒	A full description of the statistical parameters including central tendency (e.g. means) or other basic estimates (e.g. regression coefficient) AND variation (e.g. standard deviation) or associated estimates of uncertainty (e.g. confidence intervals)
☐	☒	For null hypothesis testing, the test statistic (e.g. *F*, *t*, *r*) with confidence intervals, effect sizes, degrees of freedom and *P* value noted<br>*Give P values as exact values whenever suitable.*
☐	☒	For Bayesian analysis, information on the choice of priors and Markov chain Monte Carlo settings
☐	☒	For hierarchical and complex designs, identification of the appropriate level for tests and full reporting of outcomes
☐	☒	Estimates of effect sizes (e.g. Cohen's *d*, Pearson's *r*), indicating how they were calculated

Our web collection on statistics for biologists contains articles on many of the points above.

Software and code

Policy information about availability of computer code

Data collection: All information provided in the Methods. Code deposited at OSF: https://osf.io/vka93

Data analysis:

Software Version
Conda environment mapping:
bedtools 2.26.0 and 2.30.0
hisat2 2.2.1 installed separately, compiled with `make USE_SRA=1` and $NCBI_NGS_DIR, $NCBI_VDB_DIR set as per HISAT2 manual
pandas 1.1.3
python3 3.6
samtools 1.11 and 1.16.1
sratools 2.10.9

Conda environment cafe:
cafe 4.2.1
diamond 0.9.19
mcl 14.137
python 2.7
r8s 1.80

Conda environment phylogeny:
fasttree 2.1.8
iqtree 1.6.12

muscle 5.1
mafft 7.407 and 7.511
trimal 1.4.rev15

Conda environment statistics:
R-mgcv 1.9-1

Conda environment data processing (python packages):
conda 23.7.4
python 3.7.4
basemap 1.2.1
basemap-data 1.3.2
biopython 1.78
jupyter 1.0.0
matplotlib 3.5.3
numpy 1.24.3
oceanviews (included here)
pandas 1.1.3
scipy 1.5.2
seaborn 0.11.0
statsmodels 0.12.0

Genome assembly and gene annotation:
ARACHNE 1.0
AUGUSTUS 3.3.3
BLAST+ 2.5.0
BLAT 35
BWA 0.7.8
combest 2015
exonerate 2.4.0
FGENESH / FGENESH+ 3.1.1
GeneMark-ES 2.1
GeneWise 4.0 2010-09-01
GSNAP 2019-09-12
InterProScan 5.39-77.0
MECAT 1.0
PASA 2.0.2
PERTRAN -
RepeatMasker 4.1.2 and v2002-07-13
RepeatModeler 2.0.4
RepeatScout 1.0.5
SMRT Analysis 2.2.0 (includes Quiver)
SMRT Link 4.0 (includes Arrow)

R and R packages:
R 2023.09.0+463
mgcv 1.9
ancombc 2.9.1
ASC 0.1.4
QIIME2 2019.10

Other software:
BEAST 2.2.1
BLAST+ 2.13.0+
Bowtie2 2.5
CLC Bio Genomics 21.0.3 (Server)
DIAMOND BLAST 0.9.30.131 and 2.0.14.152
eggNOG-mapper 2.1.10
FragGeneScan 1.16
HH-suite 3.3.0
HMMer 3.3.2
InterProScan 5.57-90.0
MASH-ANI 2.3
MCL 14-137
MEGAHIT 1.2.9
MFannot accessed 15/11/2020
mmseqs2 14
OGDraw accessed 15/11/2020
OrthoFinder 2.3.11
PhyloFisher 1.1.0
PROMALS3D accessed 13/1/2023
RDP4 4.101
RepeatMasker 4.0.7
REPET 3.0 (includes TEdenovo, PASTEC. TEannot)
Ribopicker 0.4.3
sDUST 0.1

Tandem Repeats Finder 4.04
Tracer 1.7.1
TreeAnnotator 2.6.4
ViralRecall 1

databases:
Pfam 35.0
PANTHER 15.0
TIGRFAM 15.0
EggNOG 5
PhyloDB 1.076
PR2 4.12.0 and 4.13.0
RepBase 25.03
RFAM 14.1
SILVA 138
UniRef30/90 release-2021_04

For manuscripts utilizing custom algorithms or software that are central to the research but not yet described in published literature, software must be made available to editors and reviewers. We strongly encourage code deposition in a community repository (e.g. GitHub). See the Nature Portfolio guidelines for submitting code & software for further information.

Data

Policy information about availability of data

All manuscripts must include a data availability statement. This statement should provide the following information, where applicable:

- Accession codes, unique identifiers, or web links for publicly available datasets
- A description of any restrictions on data availability
- For clinical datasets or third party data, please ensure that the statement adheres to our policy

Data deposited at PhycoCosm (https://phycocosm.jgi.doe.gov/Phaant1/, https://phycocosm.jgi.doe.gov/Phacord1/, https://phycocosm.jgi.doe.gov/Phaglo1/), NCBI (BioProjects PRJNA890306 and PRJNA1088233) and OSF (https://osf.io/vka93).

Research involving human participants, their data, or biological material

Policy information about studies with human participants or human data. See also policy information about sex, gender (identity/presentation), and sexual orientation and race, ethnicity and racism.

Reporting on sex and gender	*Use the terms sex (biological attribute) and gender (shaped by social and cultural circumstances) carefully in order to avoid confusing both terms. Indicate if findings apply to only one sex or gender; describe whether sex and gender were considered in study design; whether sex and/or gender was determined based on self-reporting or assigned and methods used. Provide in the source data disaggregated sex and gender data, where this information has been collected, and if consent has been obtained for sharing of individual-level data; provide overall numbers in this Reporting Summary. Please state if this information has not been collected. Report sex- and gender-based analyses where performed, justify reasons for lack of sex- and gender-based analysis.*
Reporting on race, ethnicity, or other socially relevant groupings	*Please specify the socially constructed or socially relevant categorization variable(s) used in your manuscript and explain why they were used. Please note that such variables should not be used as proxies for other socially constructed/relevant variables (for example, race or ethnicity should not be used as a proxy for socioeconomic status). Provide clear definitions of the relevant terms used, how they were provided (by the participants/respondents, the researchers, or third parties), and the method(s) used to classify people into the different categories (e.g. self-report, census or administrative data, social media data, etc.) Please provide details about how you controlled for confounding variables in your analyses.*
Population characteristics	*Describe the covariate-relevant population characteristics of the human research participants (e.g. age, genotypic information, past and current diagnosis and treatment categories). If you filled out the behavioural & social sciences study design questions and have nothing to add here, write "See above."*
Recruitment	*Describe how participants were recruited. Outline any potential self-selection bias or other biases that may be present and how these are likely to impact results.*
Ethics oversight	*Identify the organization(s) that approved the study protocol.*

Note that full information on the approval of the study protocol must also be provided in the manuscript.

Field-specific reporting

Please select the one below that is the best fit for your research. If you are not sure, read the appropriate sections before making your selection.

☐ Life sciences ☐ Behavioural & social sciences ☒ Ecological, evolutionary & environmental sciences

For a reference copy of the document with all sections, see nature.com/documents/nr-reporting-summary-flat.pdf

Ecological, evolutionary & environmental sciences study design

All studies must disclose on these points even when the disclosure is negative.

Study description	The study describes the biogeography of a group of marine algae using environmental reads mapped to their genomes. Further, it analyzes specific gene family expansions and their relevance for environmental stresses, including iron and nitrogen starvation.
Research sample	The study uses genomic data for 13 cultured strains of Phaeocystis spp. and 35 Phaeocystis-affiliated metagenome-assembled genomes (MAGs), broadly covering the diversity of these ecologically important marine algae with cosmopolitan global occurrence. We use metasequencing data from 323 stations (1573 samples; 103 metagenomic, 220 metatranscriptomic samples from Tara Oceans, Atlantic pole-to-pole transect, CICLOPS and CalCOFI-NCOG) to assess the biogeography of these genomes and specific gene expression changes in response to varying macro- and micronutrient concentrations. We also search for region-specific adaptations manifesting as Pfam expansion and expression. Finally, pilot transcriptomic data were used for P. globosa Pg-G(A).
Sampling strategy	The samples were collected or selected to capture most global marine environments (Tara Oceans, Atlantic pole-to-pole transect) as well as a level of regional diversity (CICLOPS, CalCOFI-NCOG). Genome-sequencing aimed at covering the diversity of the genus. The P. globosa transcriptomes are based on culture experiments.
Data collection	Samples were collected at marine cruises and size fractionated (see Supplementary Table S3). Standardized nucleic acid extraction, library construction and sequencing protocols were employed.
Timing and spatial scale	Tara Oceans samples were collected during multiple cruises 2009-09 to 2013-10; Atlantic pole-to-pole samples were collected during four cruises 2011-04 to 2017-01 (see original works). CICLOPS samples were collected 2017-12 to 2018-03 during austral summer. CalCOFI-NCOG samples were collected during multiple cruises 2014-02 to 2020-10 in a variety of seasons. Genomic DNA was collected in 2010 (P. globosa Pg-G(A)), 2012 (P. antarctica), and 2017 (other strains). P. globosa Pg-G(A) transcriptome samples were collected in 2002.
Data exclusions	Samples below the photic zone were excluded from this study.
Reproducibility	The analyses were performed with published data and the code is available.
Randomization	Environmental samples were for various analyses grouped by: a) station, b) depth, c) size fraction, d) oceanic domain, e) biome (temperate, Arctic, Southern Ocean).
Blinding	Blinding is not applicable.

Did the study involve field work? ☒ Yes ☐ No

Field work, collection and transport

Field conditions	Samples were collected in various conditions. The metadata are provided in the Supplementary Data.
Location	Samples were collected in various locations. The metadata are provided in the Supplementary Data.
Access & import/export	CalCOFI samples are all collected in accordance with local, federal, and international laws. Permits were not required.
Disturbance	Disturbances were not caused by the study.

Reporting for specific materials, systems and methods

We require information from authors about some types of materials, experimental systems and methods used in many studies. Here, indicate whether each material, system or method listed is relevant to your study. If you are not sure if a list item applies to your research, read the appropriate section before selecting a response.

Materials & experimental systems

n/a	Involved in the study
☒	☐ Antibodies
☒	☐ Eukaryotic cell lines
☒	☐ Palaeontology and archaeology
☒	☐ Animals and other organisms
☒	☐ Clinical data
☒	☐ Dual use research of concern
☒	☐ Plants

Methods

n/a	Involved in the study
☒	☐ ChIP-seq
☒	☐ Flow cytometry
☒	☐ MRI-based neuroimaging

Plants

Seed stocks	Algae from CCMP and NIOZ culture collections were used in the study. Accession codes are available in the Supplementary Data 1.
Novel plant genotypes	-
Authentication	-
